# Patient experiences of treatment-resistant depression (TRD): A systematic review and qualitative meta-synthesis

**DOI:** 10.1371/journal.pmen.0000128

**Published:** 2024-11-04

**Authors:** Alexandra Cernat, Manisha Pahwa, Dima Hadid, Katrina Shen, Julia Abelson, Zainab Samaan, Amanda Ramdyal, Meredith Vanstone

**Affiliations:** 1 Health Policy PhD Program, Department of Health Research Methods, Evidence, and Impact, McMaster University, Hamilton, Canada; 2 Department of Family Medicine, McMaster University, Hamilton, Canada; 3 School of Physical and Occupational Therapy, McGill University, Montreal, Canada; 4 Department of Health Research Methods, Evidence, and Impact, McMaster University, Hamilton, Canada; 5 Department of Psychiatry and Behavioural Neurosciences, McMaster University, Hamilton, Canada; UCL: University College London, UNITED KINGDOM OF GREAT BRITAIN AND NORTHERN IRELAND

## Abstract

Treatment-resistant depression (TRD) occurs when depressive symptoms persist after a patient has tried at least two antidepressants at an appropriate dose for an adequate length of time, as judged by their clinician. Understanding what it is like to live with and seek care for TRD can inform treatment planning and contribute to health technology assessments for depression-related therapies. Our objective was to systematically review this evidence through an investigation of qualitative literature about the experiences of people who have TRD. We searched MEDLINE, CINAHL, PsycINFO, and the Web of Science Core Collection. Publications eligible for inclusion were English-language primary empirical qualitative studies or the qualitative component of mixed methods studies examining adults’ experiences with TRD or with specific treatment options for TRD. Two reviewers independently screened and extracted data, as well as critically appraised included studies using the Standards for Reporting Qualitative Research (SRQR) instrument. This review was guided by integrative meta-synthesis. Data analysis consisted of a staged coding process similar to grounded theory. The protocol was registered in PROSPERO prior to the search (record ID: CRD42022356813). Sixteen studies were included from screening 18,568 results. Two studies focused on the overall experience of TRD and 14 explored experiences of specific treatments such as ketamine. Patients described the depressive symptoms they experienced, a sense of futility and desperation to find an effective treatment, and their experiences with new, sometimes experimental, therapies including deriving benefits beyond the clinical. Overall, few studies have examined the patient experience of TRD, reflecting the clinical complexity of this patient population. As most literature coalesced around experiences of specific treatments, greater examination of the overall experience of TRD is needed to inform clinical practice, and administrative and funding policy decisions for interventions that may be effective for this patient population.

**PROSPERO registration number:** CRD42022356813.

## Introduction

Depressive disorders are a group of conditions characterized by presence of “sad, empty, or irritable mood, accompanied by somatic and cognitive changes” that have a significant impact on one’s ability to function [1]. The most common depressive disorder is major depressive disorder (MDD) [2], commonly referred to simply as depression. In 2019, it was estimated that 2.49% of the world’s population was affected by depression, with the prevalence at that point in time being higher among people aged 20 years or older (3.28%) [2]. The ongoing COVID-19 global pandemic has had a severe toll on mental health and has led to a 27.6% increase in the global prevalence of MDD alone [3].

Treatment options for depression include biomedical approaches (i.e., pharmacotherapy), psychological approaches (i.e., psychotherapy), a combination of the two, or other somatic therapies such as exercise, diet, or light therapy [4, 5]. Antidepressants can be further categorized by generation. First-generation antidepressants (tricyclic antidepressants, TCAs, and monoamine oxidase inhibitors, MAOIs) were the first drugs developed to treat depressive symptoms and are often associated with intolerable side effects [6]. Second-generation antidepressants (selective serotonin reuptake inhibitors, SSRIs, and serotonin and norepinephrine reuptake inhibitors, SNRIs) refer to drugs developed since the 1980s that have improved efficacy and safety, as well as more tolerable side effects compared to first-generation antidepressants [6]. Clinical guidelines recommend most second-generation antidepressants as first-line treatments, which are a set of drugs identified by experts to be the standard initial treatment for patients with moderate-to-severe MDD [7].

After treatment is initiated, patients must be monitored and their response to treatment assessed [4]. Second-line or, subsequently, third-line antidepressants should only be considered in cases where one or more first-line antidepressants have been unsuccessful [7]. The treatment of depression has three main goals: response (i.e., a 50% reduction in symptoms from baseline); remission (i.e., return to a state of normal functioning with minimal symptoms); and recovery (i.e., two months of remission), though recovery is the ultimate aim [4, 8]. There are a number of screening instruments that can be used to assess treatment response, including self-rated scales like the Quick Inventory of Depressive Symptomatology (QIDS-SR) and clinician-administered scales like the Toronto Side Effects Scale that focuses on antidepressant side effects, in addition to clinical assessment and patient’s reported outcome [4]. Once an antidepressant is begun, it may take over eight weeks for patients to see a first response [4].

Approximately 53% of patients with MDD do not exhibit response and 67% do not experience remission in response to first-line antidepressants over the course of 14 weeks [9]. Lack of satisfactory response to antidepressants is referred to as *treatment resistance* [10], and though definitions of treatment-resistant depression (TRD) vary, a commonly accepted definition is failure to achieve remission after trying at least two antidepressants at an appropriate dose for an adequate length of time [11]. Some jurisdictions have adopted this as the formal definition of TRD, with experts reaching consensus and writing it into their clinical practice guidelines [12].

Studies examining the prevalence of TRD are sparse and vary widely in their estimates. For example, one study published in 2014 found the prevalence of TRD among Canadian primary care patients seeking treatment for depression was approximately 22% [13], while another published in 2021 used two large claims databases in the United States to find that the prevalence of TRD among pharmaceutically-treated patients was approximately 6% [14]. This range reflects not only the lack of consensus on a definition of TRD, but also the variety of challenges that exist in diagnosing this condition. To begin, the diagnosis of TRD is hampered by the phenomenon of *pseudo-resistance* [15]. This occurs when depression *appears* to be treatment-resistant, but the lack of response or remission can be attributed to suboptimal doses of antidepressants or discontinuing antidepressants prematurely, whether because of intolerable side effects or patient non-adherence [15]. True resistance to treatment is an issue of medication efficacy, not tolerability. Pseudo-resistance can also be attributed to misdiagnosis, or missing the co-existence of co-morbid conditions which accounts for many cases of depression that is apparently resistant to treatment [16–18]. Moreover, the patient population with TRD tends to be clinically complex, and response to treatment may be challenged by co-morbid conditions such as anxiety, personality, or substance-use disorders [15].

Treatment of depression may further be challenged by the fact that the aetiology of depression is complex with social, environmental, and genetic factors, as well as neurotransmitters beyond serotonin alone, playing a role [19–24]. Some authors have questioned the serotonin hypothesis of depression in an umbrella review [25], leading to media attention, however others argued that the review had methodological concerns [26]. Despite the challenges with the serotonin hypothesis, studies have consistently shown an improvement in depressive symptoms supported by randomized controlled trials (RCTs) and guidelines continue to provide recommendations for SSRIs as first-line pharmacotherapy [7].

Additionally, the term *treatment-resistant depression* is contentious because some argue that it implies a sort of therapeutic nihilism [15]. In fact, some clinicians prefer the term “treatment-*refractory* depression” to avoid instilling a sense of hopelessness in patients because therapeutic options for TRD do exist. One option to address unsatisfactory response to first- or second-line antidepressant treatment is augmentation: adding other medications not typically considered antidepressants [15]. Augmentation may be performed with lithium, triiodothyronine (T3, a thyroid hormone), or second-generation antipsychotics like quetiapine [15]. An alternative course of action is combining or switching classes of antidepressants, for example switching from an SSRI to a tricyclic antidepressant (TCA) [15]. In some cases, the decision may be made to add a different treatment modality altogether, such as psychotherapy, to ongoing pharmacological treatments [15].

Partial or non-response to depression treatment has several negative health, economic, and personal consequences, including greater likelihood of using the emergency department, greater likelihood of hospitalization, lower likelihood of being employed, and, if employed, greater likelihood of decreased work productivity [27]. TRD is also associated with reduced health-related quality of life [28]. However, these associations tell us little about the experiences of patients with TRD. Understanding what it is like to live with, seek care for, and undergo treatment for TRD is important as it can make the condition more understandable to physicians and other mental health professionals, and inform treatment planning [29]. Additionally, it can help incorporate an important patient perspective into health technology assessments (HTAs) used for health policy decision-making about the funding and implementation of depression-related treatments. Accordingly, the objective of this systematic literature review and qualitative meta-synthesis was to characterize and synthesize the state of existing knowledge. This review was guided by the following research question: *What are the experiences of and perspectives on TRD of people with this condition*, *including their experiences of and perspectives on seeking care*, *and undergoing treatment*?

## Methods

This systematic review and qualitative meta-synthesis was reported in accordance with the Preferred Reporting Items for Systematic Reviews and Meta-Analyses (PRISMA) 2020 statement [30] (S1 File). The protocol was registered in PROSPERO prior to the search (record ID: CRD42022356813), and later revised and updated to reflect the addition of the PsycINFO database.

### Search strategy

We searched MEDLINE, CINAHL (Cumulative Index to Nursing and Allied Health Literature), PsycINFO, and the Web of Science Core Collection for primary qualitative studies that addressed patient experiences of TRD. The databases were initially searched from 1987 to March 2023; the search was updated on February 9, 2024 to include studies published up until that date. The start date was chosen because newer options for treatment of depression became available allowing for switching of treatment if ineffective with the Food and Drug Administration’s (FDA’s) approval of the first SSRI (Prozac) in the United States (US) that year [31].

The search strategy was developed in consultation with an information scientist and was subsequently PRESS (Peer Review of Electronic Search Strategies) validated. Search terms included both controlled vocabulary (eg., Medical Subject Headings, MeSH) as well as free-text terms. Concepts captured through the keywords/search terms included: patients’ perspectives, experiences, attitudes, beliefs, values, perceptions, opinions, and preferences; treatment-resistant or refractory depression; and antidepressants, antidepressant switching, or augmentation. We used a validated filter to identify qualitative research [32]. The search strategy was first developed for Medline and subsequently translated for the other databases searched. The complete search strategy can be found in S2 File.

After we identified publications for inclusion through the electronic search of indexed databases, we engaged in hand-searching and citation-chaining, whereby we checked the reference lists of all included articles for additional studies that may be eligible for inclusion. We also checked the reference lists of previously published reviews that were identified through the electronic search and manually flagged during the title and abstract screening process, in accordance with methodological guidance [33]. Finally, we input the papers to be included into the smart software tool Connected Papers [34] to identify other related and potentially relevant studies that had not yet been captured in our search.

### Eligibility criteria and screening

Publications eligible for inclusion were English-language primary empirical qualitative studies examining patients’ experiences with or perspectives on TRD or on specific treatment options for TRD. Eligible study populations were adults aged 18 years or older who had TRD. The qualitative components of mixed method studies were eligible for inclusion. Studies in which participants with TRD also presented with one or more anxiety disorders (eg., panic disorder, specific phobias, generalized anxiety disorder, social anxiety disorder [35]) were included, as depression and anxiety are highly co-morbid and the literature suggests that both are strongly associated with the stable personality trait neuroticism [36, 37].

As there is complexity around defining and diagnosing TRD, at the title and abstract screening stage studies were judged as being about TRD if one or more of the following three criteria were met: (i) the terms “treatment-resistant depression” or “TRD” appeared in the title or abstract; (ii) study participants were described as having undergone at least two adequate but unsuccessful rounds of antidepressants; or (iii) study participants were described as experiencing depressive symptoms for 18 months or longer, despite being on treatment. This third criterion, while not reflective of clinical criteria for TRD, was used as a threshold to alert reviewers to the need for full text screening to ensure all potentially relevant papers were reviewed even if the study authors applied clinical criteria for TRD that differed from ours. As title and abstract screening proceeded, potentially eligible studies were tagged to indicate which of these criteria were met. For potentially eligible studies meeting criteria (i) or (iii), one of the objectives of full text review was to determine the authors’ definition of TRD. Only those papers where TRD was defined as failure of at least two adequate rounds of different antidepressants were eligible for inclusion. For example, some papers described their study sample as patients with TRD, but defined TRD as a failure of at least *one* adequate round of antidepressants; these papers were excluded. Conversely, if papers did not explicitly describe their study sample as patients with TRD but provided other information that suggested TRD, inclusion/exclusion was determined by applying this definition of failure of at least two adequate rounds of antidepressants.

Excluded from this review were: primary quantitative studies (n = 9); reviews; editorials, opinion or perspective pieces, or commentaries (n = 3); protocols or papers reporting preliminary findings without including sufficient empirical data (n = 1); conference abstracts, posters, or presentations; clinical practice guidelines; and grey literature including articles published in the News section of scientific journals. Unpublished works such as theses or pre-prints were also excluded (n = 1). Studies that exclusively used a quantitative scale to evaluate patient experiences were excluded as they are closed-ended and necessarily predefine aspects of patient experience. Studies that did not focus on patient perspectives were excluded (n = 5).

Studies were excluded on the basis of their included patient population (n = 88). In particular, studies that incorporated any pediatric participants were excluded, as were studies that focused on depression not described as treatment-resistant or those in which participants’ primary diagnosis was unclear. Studies about chronic, persistent, or recurrent depression were also excluded if there was no indication that participants experienced failure of at least two adequate antidepressant rounds, as these are forms of depression distinct from TRD. Studies were also excluded if they focused on depression or TRD in pregnant people or those with postpartum depression, as these periods are commonly marked by a variety of drastic physiological and lifestyle changes that may result in causal attribution. Studies in which the majority of participants had Alzheimer’s disease or dementia, bipolar disorder, borderline personality disorder, post-traumatic stress disorder (PTSD), or psychotic illnesses (eg., schizophrenia, schizoaffective disorder, depression with psychotic features, drug-induced psychosis, or delusional disorder [38]) were also excluded. We also identified studies about circumstantial or situational TRD, such as that which occurred in relation to illness (eg., cancer, HIV), divorce, or death of a loved one. These papers about situational depression were excluded on the principle that it would not have been possible to untangle the experience of these illnesses or significant life events from the experience of depression. Studies about a different topic altogether were excluded (n = 1). Studies examining the clinical or cost-effectiveness of antidepressants or other treatment options for TRD were excluded. To ensure comparable health resources were available to participants in included studies, study locations were limited to high-income member countries of the Organization for Economic Co-operation and Development (OECD) or European Economic Area (EEA) (n = 1).

Titles and abstracts were screened by two independent reviewers (AC and one of MP, DH, or KS). Where we conflicted, we arrived at a final decision through discussion. Full texts were obtained for those references that met eligibility criteria, as well as for references where the title and abstract alone were insufficient to determine eligibility. The same reviewers screened the full texts, and once again, conflicts were resolved through discussion and consensus. All screening was performed in Covidence [39]. A PRISMA flow diagram was constructed to depict the flow of information through the review. A summary of the inclusion and exclusion criteria are shown in Table 1.

**Table 1:** Review inclusion and exclusion criteria.

	*Included*	*Excluded*
***Study Design***	Primary empirical qualitative research studies published in peer-reviewed journals. The qualitative component of primary mixed method studies published in peer-reviewed journals.	Primary quantitative studies. Reviews or analyses of secondary data. Editorials, opinion or perspective pieces, or commentaries. Protocols or papers reporting preliminary findings. Conference abstracts, posters, or presentations. Unpublished works (eg., theses, pre-prints of manuscripts submitted for publication). Clinical practice guidelines for antidepressant use or the treatment of depression. Grey literature, including articles published in the News section of journals.
***Population***	• Adults with TRD, defined as poor or non-response to at least two adequate rounds of antidepressants.	Children and youth aged 18 years or younger. Adults with Alzheimer’s disease or dementia, bipolar disorder, borderline personality disorder, or post-traumatic stress disorder co-morbid with TRD. Adults with depression that may be linked to a causal event or experience such as diagnosis of a chronic disease or loss of a loved one. Pregnant people or people with post-partum depression.
***Topic***	• Experiences with or perspectives on TRD or treatment options for TRD.	• Clinical or cost-effectiveness of antidepressants or other treatment options for TRD.
***Location***	• High-income OECD or EEA member countries.	Low- and middle-income countries. High-income countries not part of the OECD or EEA.
***Language***	• English.	• Languages other than English.

EEA: European Economic Area

OECD: Organization for Economic Co-operation and Development

TRD: treatment-resistant depression

### Data extraction and analysis

Data extraction was independently performed by two researchers (AC and one of MP, DH, or KS) using a data extraction form in Covidence. Extracted data included: bibliographic information, study purpose, study design and main methods, and participant characteristics. The main findings were extracted using NVivo [40]. Our data in this review consisted of the authors’ interpretations of their own findings: the “data-driven and integrated discoveries, judgements, and/or pronouncements researchers offer about the phenomena, events, or cases under investigation” [41]. Data extraction was completed November 2022-April 2024.

Analysis consisted of a staged coding process akin to that of grounded theory [42]. Included studies were broken into their conceptual components; these were summarized and regrouped based on their thematic relationships. We first performed line-by-line coding to identify preliminary categories, which were established based on both the prevalence of information across included studies as well as the relevance of that information to our research question. The primary author (AC) met with other members of the research team (MV and MP) to discuss these categories and decide which should be the primary focus as analysis proceeded. Broader themes emerged through each subsequent round of coding. We used a constant and iterative approach, comparing the categories we developed with the findings of the papers themselves and our interpretations of those papers. Overall, this approach enabled us to reflect “the range of findings, while retaining the original meaning of each study, offering a new integrative interpretation which both describes findings across the studies and interprets meaning from the collective body of literature” [43].

### Critical appraisal

We critically appraised all studies included in this review using the Standards for Reporting Qualitative Research (SRQR) instrument [44]. This is a tool that balances brevity and comprehensiveness, was developed through both a literature review and expert opinion, and applies to all qualitative methodologies [45]. No studies were excluded on the basis of this critical appraisal since methodological and procedural details may often be omitted from a manuscript despite their presence in the research process [45]. We re-created the SRQR instrument in Covidence and critical appraisal of all included studies was performed by two independent reviewers (AC and one of MP, DH, or KS) within that platform. Where we conflicted, we reached a final decision through discussion.

## Results

**Fig 1:**
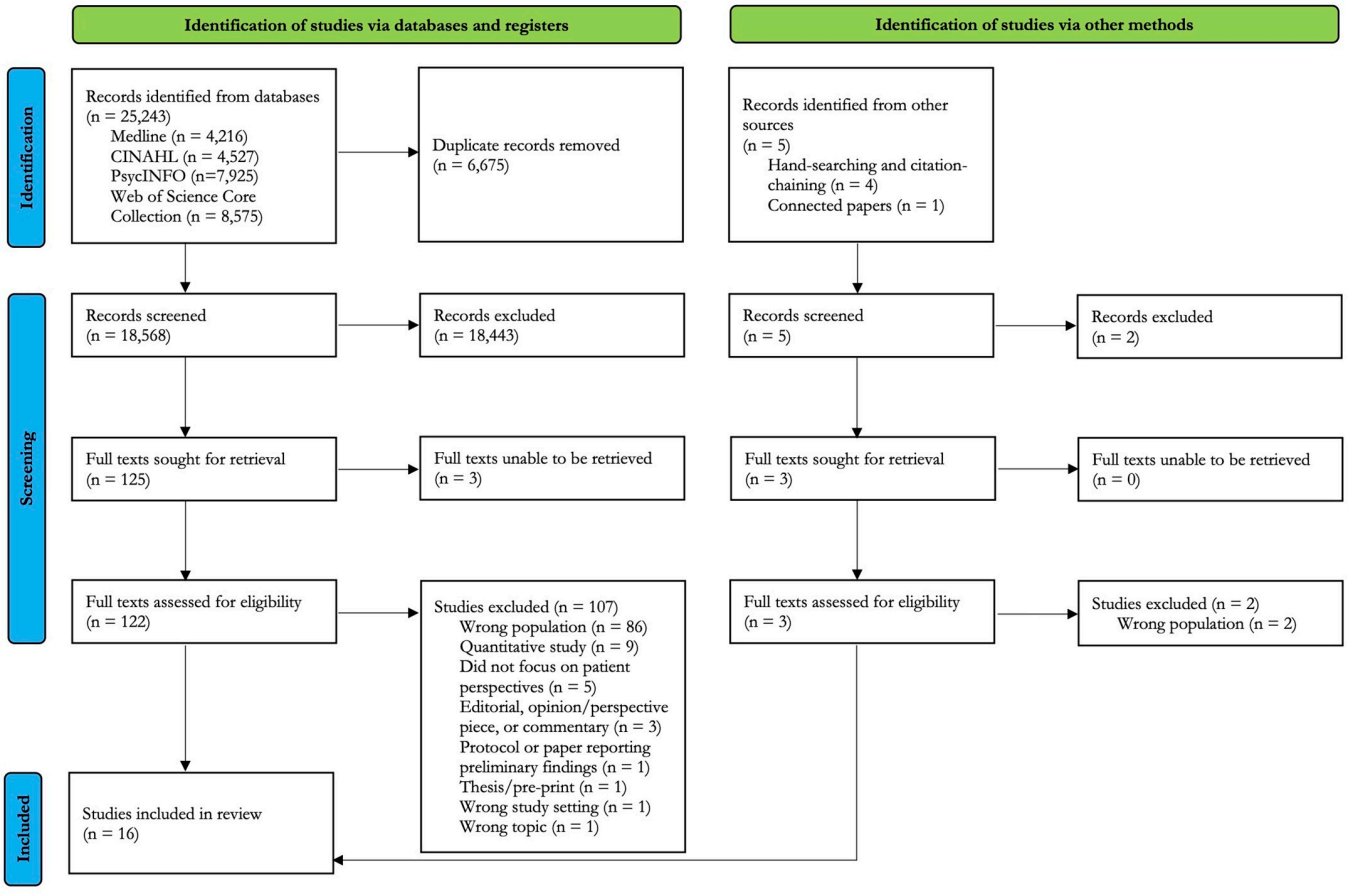
PRISMA flow diagram.

A total of 18,568 references (duplicates removed) were retrieved using the electronic database search strategy. Of those, 125 were deemed potentially eligible for inclusion and proceeded to full-text review. We were unable to access the full texts of three references, so only 122 full texts were assessed for eligibility. An additional five papers were identified through hand-searching and citation-chaining, of which two were excluded in title and abstract screening and two were deemed ineligible in full-text review. One additional record was found through Connected Papers but was excluded. The search is depicted in the PRISMA flow diagram shown in Fig 1. In total, 16 primary qualitative studies were included [46–61]. A list of the full texts that were reviewed but ultimately excluded is provided in S3 File with the reason for exclusion for each paper. A list of all references retrieved but screened as irrelevant is provided in S4 File. Eighty-four out of the 125 full texts we reviewed (retrieved through the electronic search, hand-searching, and citation-chaining) were excluded because participants had MDD but not TRD, or there was insufficient information to determine whether participants had a treatment-resistant form of MDD.

The 16 included papers included a total of 242 participants, of whom 201 (83.1%) had unipolar TRD. The forty-one individuals across all studies who did not have TRD had MDD but not TRD (12) [50], bipolar disorder (11) [46, 47, 49, 53, 54, 56], and emotionally unstable personality disorder (2) [53]. Eleven participants had been diagnosed with bipolar disorder but met criteria for TRD and had been experiencing depressive symptoms continuously for at least one year [55], and five participants were caregivers [59, 60]. Wherever possible, data attributed to participants who did not fulfil the TRD criteria were separated and excluded from this review. Participant sex or gender was reported for 216 of 242 individuals; 114 (52.8%) were described as female/women and 102 (47.2%) were described as male/men. Participant ages ranged from 18 years [51] to 77 years [55], though not all studies [56, 58] provided an age range. Six studies reported the race or ethnicity of their participants [49, 52, 53, 55, 57, 61]. Across those six papers, there were 102 participants, of whom 59 were white, eight were Black, one was Asian, one was Hispanic, nine were described as New Zealand European, and one was described as “other” meaning not white British. Lawrence et al. [55] only reported the race or ethnicity of five of their 24 participants, stating that five participants were non-white and that zero participants were Hispanic or Latino. Watts and colleagues [61] reported the race or ethnicity of 20 individuals although only 19 ultimately participated in qualitative interviews. With a few exceptions (for example [55, 59]), studies did not report further demographic information such as participants’ average income, education level, or employment status. While sexual orientation has been found to be associated with depression [62], none of the included studies reported information about participants’ sexual orientation.

Few studies reported the length of time since the onset of participants’ depression [51, 54, 58–60] or the number of antidepressants tried by their participants [47, 58]. Some participants reported decades-long experience with depression [51, 54]. Table 2 describes the studies captured in this review, including the study design, main purpose or research question addressed, geographic location, and participant characteristics.

**Table 2:** Summary of included studies.

Author (Year)	Country	Main Purpose	Qualitative Methodology	Analytic Approach	Participant Information
**Breeksema et al. (2022)** [46]	The Netherlands	To understand the experiences of patients with TRD with esketamine treatment and understand the treatment conditions which may facilitate the best outcomes.	Interpretative phenomenological analysis.	Interpretative phenomenological analysis.	Seventeen participants in total. Fourteen participants had TRD, defined as experiencing insufficient response to adequate treatment with at least three different classes of antidepressants. Three participants had a primary diagnosis of bipolar disorder. Five of the participants with TRD had co-morbidities: two had autism spectrum disorder; one had personality disorder, not otherwise specified; one had avoidant personality disorder as well as symptoms of PTSD; and one had both autism spectrum disorder and PTSD.
**Breeksema et al. (2023)** [47]	The Netherlands	To understand the experiences of patients with TRD with oral esketamine therapy.	Interpretative phenomenological analysis.	Interpretative phenomenological analysis.	The same participants as in Breeksema et al. (2022) [46], described above. Additional information about participants was reported in this study, specifically that the mean number of antidepressants tried across all 17 participants was 9.3 and the mean length of the current depressive episode was 55 months.
**Breeksema et al. (2024)** [48]	The Netherlands	To examine the experiences of people who participated in a clinical trial comparing oral psilocybin therapy with placebo for the treatment of TRD.	Interpretative phenomenological analysis.	Interpretative phenomenological analysis.	Eleven participants with TRD, which the authors defined as a “failure to respond to at least two antidepressants with adequate doses and durations.” No co-morbidities were reported. Length of current depressive episode, time since onset of depression, and mean number of antidepressants tried by the participants were not reported.
**Griffiths et al. (2021)** [49]	United Kingdom	To understand patients’ experiences of ketamine treatment for depression.	Interpretative phenomenological analysis.	Interpretative phenomenological analysis.	Thirteen participants in total. Eleven participants had TRD, which the authors defined as a failure of at least two antidepressants. Two participants had bipolar affective disorder. One participant with TRD had co-morbid mixed anxiety disorder and one participant with TRD had co-morbid schizoaffective disorder.
**Kerr et al. (2023)** [50]	United Kingdom (England)	To examine the lived experiences of people with symptomatic depression, symptomatic TRD, or TRD in remission.	Not specified.	Thematic hybrid deductive and inductive approach.	Twelve participants had symptomatic TRD, 12 participants had symptomatic MDD, and two participants had TRD that was in remission. No participants had ever been diagnosed with a neurodegenerative or behavioural/personality disorder, or another mental disorder such as bipolar disorder. None had a co-morbid psychotic disorder, depression with psychotic features, or OCD.
**Kragh et al. (2017)** [51]	Denmark	To understand patients’ experiences with wake and light therapy and understand factors that may affect adherence to therapy.	Not specified.	Qualitative content analysis.	Thirteen participants with TRD who were described as having mild to severe resistance to treatment according to the Maudsley Staging Method. Length of time since onset of depression ranged from less than one year to 21 years.
**Kroch et al. (2022)** [52]	New Zealand	To examine how people with TRD understand and make sense of their depression.	Narrative.	Narrative analysis.	Nine participants with MDD who had tried at least two antidepressants from different classes and did not experience significant clinical improvement.
**Lascelles et al. (2019)** [53]	United Kingdom	To understand the effect of ketamine treatment on suicidal ideation in patients with TRD.	Not specified.	Thematic analysis.	Fourteen participants in total. Ten participants had TRD. Two participants had a primary diagnosis of bipolar affective disorder type 1 and two had a primary diagnosis of emotionally unstable personality disorder.
**Lascelles et al. (2020)** [54]	United Kingdom	To explore patients’ with TRD experiences of and perspectives on ketamine treatment.	Not specified.	Thematic analysis.	Twelve participants in total. Eleven participants had TRD and had tried at least two antidepressants at an adequate treatment dose for six weeks each as well as at least one type of psychological treatment. One participant had bipolar disorder type 1. Length of time since onset of depression ranged from 10 to 50 years. One participant had co-morbid OCD.
**Lawrence et al. (2018)** [55]	United States	To explore the perspectives, beliefs, and values of patients with TRD with respect to deep brain stimulation.	Not specified.	Not specified.	Twenty-four participants in total, 13 of which had unipolar depression and 11 who had “ever been” diagnosed with bipolar disorder. Although no distinction between these two categories of participant were made in the results of this paper, this study was still included as all 24 participants met criteria for TRD, specifically that they had tried five or more pharmacological treatments for depression. All participants, including those who had at some point received a bipolar disorder diagnosis, had been experiencing depressive symptoms continuously for at least one year. The mean length of participants’ current depressive episode across the study sample was 2.1 years.
**Raffin Bouchal et al. (2023)** [56]	Canada	To explore processes of personal recovery among patients with TRD after deep brain stimulation.	Constructivist grounded theory.	Grounded theory (i.e., staged coding, constant comparison).	Eighteen participants in total. Fifteen participants had TRD. Three participants had a primary diagnosis of bipolar disorder.
**Starr et al. (2020)** [57]	United States	To examine patients’ experiences with esketamine treatment and elicit their overall perspectives on this therapy.	Not specified.	Constant comparative analysis.	Twenty-three participants with TRD who had responded to treatment and who were participating in an open-label safety study of esketamine + oral antidepressant treatment.
**Sumner et al. (2021)** [58]	New Zealand	To examine patients’ perspectives on the effect ketamine treatment had on their depressive symptoms, and understand the impact of patients’ participation in a ketamine trial on their perception of their depression and likelihood of pursuing further treatment in the future.	Not specified.	Thematic analysis.	Thirty-two participants with MDD who experienced an inadequate response to at least two antidepressants. On average, participants had unsuccessfully tried 3.5 antidepressants. Although data is provided for all 32 participants, only 31 participated in qualitative interviews. Twenty-three participants reported co-morbid anxiety.
**Thomson et al. (2021)** [59]	Australia	To understand the experiences of patients with TRD and their caregivers with deep brain stimulation.	Not specified.	Thematic analysis.	Eleven participants in total. Six participants were patients with TRD. Five participants were caregivers of patients with TRD. The mean time since diagnosis of depression was 18.3 years.
**Thomson et al. (2023)** [60]	Australia	To understand the experiences of patients with TRD and their caregivers before and after deep brain stimulation.	Not specified.	Thematic analysis.	The same participants as in Thomson et al. (2021) [59] described above.
**Watts et al. (2017)** [61]	United Kingdom	To qualitatively analyze the effects of psychedelic treatment on patients with depression in a modern controlled trial.	Phenomenology.	Thematic analysis.	Nineteen participants with TRD who had tried at least two antidepressants without exhibiting response.

MDD: major depressive disorder

OCD: obsessive-compulsive disorder

PTSD: post-traumatic stress disorder

TRD: treatment-resistant depression

The results of the critical appraisal are presented in S5 File. Studies were considered of acceptable quality. In all studies, the main findings were described in great detail with links to empirical data such as participant quotes. In addition, all studies integrated their main results with prior work and described the implications of their findings. Limitations were identified in all 16 included papers. Data collection methods were described in adequate detail in the majority of studies and data collection instruments (i.e., the interview guide) were well-described in 15 of the 16 included papers ([57] being the exception). All papers except [48, 51] provided adequate detail with respect to data analysis methods. However, only seven studies [46–49, 52, 56, 61] identified a specific qualitative approach. Only one paper made mention of reflexivity [52] but none included any formal reflexivity statement. The sampling strategy was not described in eight of the included papers [51–54, 56–58, 61], and only one study provided sufficient detail when describing their participants [47].

Of the included papers, only two focused on the overall experience of TRD [50, 52], while 14 examined experiences of specific treatments (ketamine/esketamine, deep brain stimulation (DBS), psilocybin, and wake and light therapy) [46–49, 51, 53–61]. In the therapy-specific papers, nearly all participants were naïve to the treatment in question. In most cases, the participants were enrolled in a trial examining the efficacy of the intervention [48, 51, 56–61]. In the sections below, when we refer to a “new therapy,” we mean the treatment that participants were trying for the first time in the therapy-specific studies, rather than a novel treatment.

Although these papers had heterogeneous aims, there were commonalities in the descriptions of experiences of TRD and new therapies that enabled us to identify themes or categories of findings belonging to three main groups: i) symptoms of depression; ii) response to past treatment failures; iii) experiences with a new therapy.

### Symptoms of depression

Participants likened depression to being “trapped” [47] in a “mental prison” [61] and described their experience as a “heaviness, darkness, hopelessness, bleakness, psychic pain, a diminishing world, or being submersed in a swamp” [47]. A process of “rumination” [47, 61] seemed to be at the centre of depression, whereby participants constantly became stuck on negative thoughts that whittled their self-confidence and impeded their ability to partake in activities they had once enjoyed [47, 53, 61]. They felt core aspects of their personalities and identities had become suppressed [60], and lacked motivation or energy to engage in activities of daily functioning such as cooking, cleaning, or otherwise taking care of themselves [50, 57]. At the same time, participants reported trying to prevent thinking about painful memories, particularly those related to traumatic events they believed contributed to the development of their depression [61]. Moreover, participants described physical and cognitive problems like difficulty sleeping, sound sensitivity, and impaired memory and concentration [50, 51, 57].

In addition to feeling disconnected from themselves, participants also felt disconnected from others and the world around them [47–50, 61]. Participants reported feeling isolated, lacking bandwidth or interest to invest in personal connections, and characterized their relationships with family and friends as strained [47, 49–51, 61]. In one study, patients with TRD and their caregivers were described as “maintain[ing] strong and meaningful bonds” even though depression had a negative impact on their interpersonal dynamics [60].

### Response to past treatment failures

#### Futility: The return of depression is inevitable

Participants described depression as both predictable and unpredictable [52]. For instance, some could identify patterns within their depressive episodes (eg., waking up feeling low, improving during the day, and waking up the next morning worse again), and others described early depressive episodes as being clearly triggered by an external factor or event such as a birth, death, ending of a relationship, and so on [52]. However, as time passed, it seemed as though the onset of depressive episodes was less and less related to identifiable stressors [52]. Eventually, participants simply “gave up” trying to think about their depression as a logical or predictable phenomenon, and instead began to describe themselves as somehow intrinsically different than people with depression who respond to treatment [52]. Because they had been experiencing depressive episodes for months or years, many participants no longer believed recovery was possible, and some expected or worried their symptoms would return even in periods where they were feeling relatively well [50–52, 56]. Some also expressed disappointment in the mental healthcare system, and a lack of trust or faith that their mental healthcare providers would listen to them [48, 50].

The sense that the return of depression was inevitable coloured the way participants approached therapies they had never tried before. For example, participants described suppressing expectations that a new treatment would work to avoid disappointment if it was ineffective [46, 48, 54, 58, 59]. Others described being hopeful that their new therapy would work but tempering that hope with the knowledge that all the treatments they had tried in the past had been unsuccessful [51, 54] or that their clinician had explained the new therapy would not “be the solution” that would “get rid of [their] depression” [46]. One study found that half of participants asked felt there was less than a 50% chance they would benefit from deep brain stimulation (DBS) and half thought there was a 50% chance or greater [55].

Despite their “cautious optimism” [51, 56], participants felt despair, hopelessness, disappointment, and frustration when therapies did not work for them or failed to meet whatever expectations they did have, or did not work as quickly as they expected [50, 51, 54, 58, 61]. For some, these feelings were so strong they led to suicidal thoughts, wherein participants questioned the purpose of being alive if they would never experience sustained improvements in their mood and wellbeing [51, 54]. These participants blamed themselves for treatments repeatedly not working, expressed apprehension at the thought of having to seek out yet another treatment option that they had not yet attempted, and described an overall feeling of defeat [54, 58].

#### Desperate desire to get better

Participants’ belief that attempts at recovery were futile were largely borne out of their past experiences with treatment. Participants had tried a wide range of therapies—antidepressants, psychotherapy, electroconvulsive therapy (ECT), repetitive transcranial magnetic stimulation (rTMS), diet and exercise, herbal medicine—all with limited and unsatisfactory effect [46–48, 51, 54]. Participants across studies had all tried a variety of antidepressants, often trying multiple medications at once, but did not experience improvement in their symptoms, experienced only temporary relief, or experienced severe side effects that prevented continuation of that therapy [46, 47, 50–52, 57, 61]. Willingness to continue trying new treatments illustrated how desperate patients were to get better. At the same time, participants’ long histories of trying treatment after treatment without success also reinforced their desire for depression recovery and made them even more desperate, as they were losing hope they would ever find something that works well for them [51].

In some studies, participants explicitly described themselves as being “desperate” [49, 51] for something to alleviate their depression, and described therapies such as esketamine or DBS as “extreme” [59] treatments of “last resort” [46, 49, 56, 59] that they were trying or would only consider because they had exhausted all other options [46, 49, 55, 56, 59]. One participant described trying esketamine within a limited timeframe, after which if there was no improvement in their depression they would pursue euthanasia [46]. Although some patients were not interested in DBS at all, desperation for an effective treatment was observed among others who declared an intention to tolerate risks of serious adverse events [55].

Patients’ willingness to try other therapies not supported by a strong or well-established evidence base was also interpreted as related to a desperate desire for symptom improvement even when it was not explicitly framed in this way. For example, some of the patients who tried psilocybin were taking part in the first ever clinical trial of this therapy for the treatment of TRD [61]. A desperate desire for improvement was also identified in participants’ response to challenging or unpleasant experiences while taking part in a trial. Participants trying light therapy found that staying awake for 36 hours at a time was difficult, experiencing physical discomforts like headache, shivering, heartburn, but also mental challenges as they felt they became more sensitive and vulnerable [51]. Even so, participants’ desire for symptom improvement was an overriding consideration and led to increased adherence, as “giving up was not an option” [51].

### Experiences with new therapies

#### New therapies as intense or overwhelming

Participants often described having “intense” [46, 48, 61] or “overwhelming” [46, 48] experiences or emotions during sessions with new therapies, particularly ketamine/esketamine and psilocybin. These were sometimes characterized as dissociation, becoming detached from their bodies, a distortion of time and space, or feeling as though they had entered a different world or dimension [46, 47, 49, 53, 58]. Some of these experiences were positive, but they could also be “disorienting [and] alienating” [46],“frightening” [46, 47], or otherwise negative [53, 58].

#### Impact on depression and side effects

Many participants in trials of new therapies reported that their symptoms improved, though the effects of treatment tended to be temporary or transient [47, 49, 53, 54, 56–59, 61]. In particular, participants reported improved mood, that their negative thoughts became interrupted, making room for more positive thoughts; that they had clarity of thought and improved focus; and that they felt “normal” or back to their old selves [47, 49, 51, 53, 54, 56, 57, 59–61]. Some described a reduction in their suicidal ideation [49, 53, 54, 57]. In general, these improvements enabled participants to reconnect with friends and family and engage in conversations; take part in day-to-day activities like housecleaning; make positive lifestyle changes like exercising regularly and eating healthier; and resume leisurely activities they had once enjoyed or try new activities [47, 49, 53, 54, 56–61].

At the same time, some participants only reported mild improvements with their new therapy while others did not respond at all [49, 51, 54, 56, 58, 60, 61]. Some also reported side effects or even a worsening of their depression [47, 49, 53, 54, 56–59] which in some cases were so severe that participants had to discontinue treatment [53, 54]. Even so, overall participants felt that the new therapies, particularly esketamine and psilocybin, were superior to treatments they had tried in the past [57, 61].

#### Deriving benefit from antidepressant alternatives beyond the clinical

Beyond symptom improvement, having the opportunity to participate in a trial or try a treatment they had not yet explored seemed to help combat some of the feelings of futility and treatment nihilism that had previously been described. To begin, trying a new therapy renewed participants’ hope and confidence in the possibility of recovery [51, 58]. When participants experienced symptom improvement, they reported feeling hopeful for the future, because now there was a possibility they would get better that had not existed before [53, 54, 58]. Their positive experience with the new therapy also motivated them to continue to try other treatments they had not tried before after the conclusion of the trial they were currently participating in [58]. Even when the new therapy did not work, participants still felt that trying it had been worthwhile, were grateful to have had the option of trying an alternative to antidepressants, and felt that others should have the opportunity to try the new therapy [49, 51, 54, 59, 61].

## Discussion

Patients with TRD described their depressive symptoms, and emphasized how these symptoms contributed to experiences of isolation [47, 49–51, 61], hopelessness [47], and self-blame [54, 58]. Patients reported believing they will never recover from depression, as well as being desperate to find an effective treatment, which impacted their experiences with new, sometimes experimental, therapies. Patients often approached new therapies with “cautious optimism” [51, 56], lowering their expectations in an attempt to avoid disappointment if the new therapy proved to be yet another ineffective treatment [50, 51, 54, 58, 61]. Some participants likened depression to a “prison” [61] of the mind, a term that has previously been used in the literature to describe the experience of this condition [63]. These findings share similarities with the experiences of people who have depression not classified as resistant to treatment. For instance, patients with treatment-responsive MDD felt hopeless while in the middle of a depressive episode, were unsatisfied with their antidepressant treatment and reported trying multiple medications before finding one that worked well, and described feeling frustrated or desperate if their antidepressant did not adequately relieve their symptoms [64–66]. However, the experiences of people with TRD and those of people with MDD are also different in several important respects. To begin, the hopelessness felt by patients with MDD was described as a “bothersome symptom” [64] experienced when feeling depressed, as opposed to a general state of being that persisted even outside of depressive episodes in people with TRD. Furthermore, unlike participants with TRD, the majority of patients with MDD did consider their antidepressants to be effective, particularly in reducing their anxiety, making them feel less overwhelmed, increasing their motivation and optimism, making them feel better able to socialize, and overall helping them maintain a normal life [64, 66]. Their dissatisfaction stemmed instead from the experience of side effects, perception that their antidepressants did not take effect quickly enough, lack of desire to be on long-term medication, and, in some cases, a lack of relief from the symptom of fatigue specifically [64, 66].

This work has implications for health technology assessment (HTA) and policy-making as patient experiences, perspectives, attitudes, beliefs, values, and preferences are increasingly being incorporated into decisions about the development, implementation, and funding of drugs and health technologies. HTA bodies, both in Canada and internationally, recognize the importance of this type of evidence [67, 68]. For instance, a recent HTA of pharmacogenomic (PGx) testing in depression care included direct engagement with patients with depression, speaking to both medication-naïve individuals, as well as to people who did not adequately respond or were unable to tolerate at least one drug [69]. Findings from that exercise affirm findings of futility and desperation in the present review, underscoring the importance of engaging with this patient population when prioritizing and assessing depression-related services and technologies [70–72]. Understanding the feelings of desperation experienced by patients who have exhausted all traditional therapies may influence the policy consideration of “atypical” or less “traditional” therapies for this population.

Furthermore, these findings may also have implications for clinical practice and research relating to new interventions, particularly those which may be considered “extreme” [59] or of “last resort” [46, 49, 56, 59]. Existing literature highlights ethical challenges associated with offering or withholding such treatments for patients with TRD [70–73]. For patients and families who are desperate to achieve remission after exhausting numerous treatment options, being offered the opportunity to try an alternative therapy or intervention (eg., hospital admission, psychotherapy, medication, neurostimulation), even one with limited expected benefit, may foster hope, though only about one-fifth of psychiatrists report they would provide additional neurostimulation specifically (i.e., transcranial magnetic stimulation, electroconvulsive therapy, etc.) to patients with depression if they believed this type of treatment would ultimately be unhelpful [70]. At the same time, others have argued that applying the label of “medical futility” without having explored the full range of available treatments can have negative consequences for patients [71]. In the research context, concerns have been raised about informed decision making with respect to deep brain stimulation (DBS) for depression [73, 74]. These stem both from patients’ desperation and vulnerability, and the fact that depression itself may impact one’s cognitive abilities [74], as well as several important differences between DBS studies and more typical research with depressed patients [73]. The procedure to implant the DBS system is an invasive brain surgery that carries risk in-and-of itself, and more than that, such research studies may be longer and more complex than trials involving pharmacotherapy due to a need for appropriate follow-up care and monitoring [73]. Consequently, there has been discussion in the literature as to whether additional safeguards are required to ensure that informed consent processes in DBS studies with patients with TRD are ethically sound [73]. Dunn and colleagues [73] have argued that additional safeguards beyond what would typically be needed in any high-risk research study are not necessarily required, though they highlight a paucity of empirical evidence about inherently relevant topics such as decision-making capacity among patients with TRD who are severely ill, or the effects of depression on decision-making. In any case, transparency, the setting of realistic expectations, and engaging in a robust conversation with patients and research participants about what a procedure entails, potential benefits, and potential risks are critical prior to exposing them to interventions that may cause unnecessary harm while offering unclear or limited clinical effectiveness [70, 72].

One of the main findings of this review is that there are few studies examining the experiences of patients with TRD, with this information typically included in studies focused on the experiences of particular interventions for TRD. This likely reflects clinical difficulty identifying members of this patient population. One major challenge is that the proportion of patients diagnosed with MDD who have true resistance to treatment is likely much smaller than what current estimates [13, 14] suggest because misdiagnosis is common. There is strong evidence from several countries that patients with bipolar disorder are often misdiagnosed as having MDD [75–77], and this group contributes to the phenomenon of pseudo-resistance because antidepressants for treatment of bipolar disorder tend to be ineffective and can in some cases exacerbate bipolar disorder symptoms [78]. Similarly, some patients labeled as having TRD may have unrecognized depressive subtypes such as unipolar MDD with psychotic features, for which antidepressants alone would be ineffective [7].

Beyond misdiagnosis, the identification of patients with TRD is complex due to the co-occurrence of MDD with co-morbidities such as anxiety disorder, substance abuse, personality disorders, and non-psychiatric chronic and organ disease—all of which are predictive of resistance to depression treatment [12]. Pseudo-resistance may also arise when patients are co-prescribed medications for their psychiatric or physical health that act as CYP450 (a group of enzymes associated with liver drug metabolism) inducers, as these may reduce plasma levels of some antidepressants [79]. Smoking can also influence medication metabolism, and reductions in the serum levels of fluvoxamine, duloxetine, trazadone, and mirtazapine in particular have been observed in smokers compared to non-smokers [80]. As over half of patients with MDD are lifetime smokers [81], this may be a contributing factor to treatment effectiveness and addressing the smoking status of patients with TRD could potentially enhance treatment response.

Beyond challenges identifying patients with TRD, the clinical complexity of TRD patients may also impede their participation in research. This may happen explicitly, such as when studies exclude patients with co-morbidities, and implicitly, related to the energy, organization, and effort required to offer consent and complete the required research activities. Even in cases where the presence of co-morbidities does not render patients ineligible for participation in primary empirical work, secondary work may exclude papers with clinically complex patient populations, much like we have done in the present review. Finally, it may be difficult to recruit patients with TRD as study participants: some clinicians may avoid using the term “treatment-resistant” in favour of terms such as “treatment-refractory depression” or “difficult-to-treat depression” to avoid introducing therapeutic nihilism [82], and as such some patients may not identify themselves as having TRD.

These challenges notwithstanding, it is clear there is also an issue of research reporting, particularly in qualitative research of patient experience. During the screening process of this review, we identified over 100 studies that were potentially relevant and required full text assessment to determine inclusion. Of those, the majority (84) were excluded because their participants consisted of people with MDD but not TRD, or because it was not possible to determine whether the participant population had TRD defined as failure of at least two adequate rounds of antidepressants (S3 File). For example, one paper’s study population consisted of patients who had been diagnosed with depression between 18 months and 44 years prior to study initiation, but their diagnoses were all listed as simply “depression” with no further specification and no antidepressant history described [83]. It is conceivable that at least some of these participants had TRD, but we felt it necessary to exclude it because we could not confirm that this paper met our inclusion criterion of participants having failed at least two adequate rounds of antidepressants.

The issue of inadequate or incomplete research reporting indicates there may be a need to standardize the type of information presented about research participants with heterogeneous conditions like depression, much the way certain demographic information (eg., sex or gender; race or ethnicity) is almost always presented. Information relevant to depression that authors should consider including includes the time since onset of participants’ depression, the length of the current depressive episode or average length of participants’ depressive episodes, frequency of episodes, and the number of antidepressants that participants have tried but found to be ineffective. This information might help identify participants as having a specific type of depression regardless of how authors choose to describe their study population. Of course, caution is needed to ensure that none of this information is presented in a way that could identify participants or jeopardize their privacy.

### Strengths and limitations

There is one existing systematic review on this topic [84] with nine included studies. That review used a search strategy that consisted of nine terms, yielding 1,148 references. In contrast, our search strategy was much more comprehensive. We retrieved 25,243 references from four databases. We supplemented this strategy with hand-searching and citation-chaining. The rigour of our review was supplemented with the use of a consistent, clinically validated and commonly accepted definition of TRD (failure of two or more adequate antidepressant rounds) to assess whether potentially eligible papers were about this condition or another type of depression. Our rigour in applying this definition led us to exclude seven of the nine papers included in the other review [84] because we could not determine if they met this criterion for TRD. Ultimately, only two papers [52, 61] overlap between [84] and the present review.

The findings of this literature review must be interpreted in the context of several limitations. We only included papers written in English. Since studies conducted in high-income member countries of the OECD or EEA (including those where English is not a primary language) were eligible, this restriction means we may have excluded otherwise eligible papers in which qualitative interviews were conducted in a language other than English. Moreover, we excluded papers in which the majority of participants had psychiatric co-morbidities other than anxiety. While we acknowledge that such co-morbidities are common and therefore that some studies otherwise eligible may have been excluded, we felt it was important to establish this criterion given the contribution of co-morbidities to the phenomenon of pseudo-resistance. Additionally, qualitative research findings are not intended to be generalized to entire populations. As all of the papers included were conducted in high-income countries with predominantly white participants, the insights from this review may not be transferable to other contexts.

## Conclusions

This review synthesized existing qualitative research about patients’ perspectives of and experiences with TRD. Sixteen papers were included, of which 14 focused on experiences of specific therapies. Patients described depression as a state of disconnection and felt an overall sense of futility and hopelessness that they would ever fully recover. At the same time, they were desperate to get better and were hopeful that new treatments may provide relief, though this hope was tempered by their history of recurrent depressive symptoms. Greater examination of the overall experience of TRD is required, particularly if evidence about patient perspectives, experiences, attitudes, values, and beliefs is to be used to inform clinical practice and policy decision-making, particularly with respect to administrative/implementation and funding decisions.

## Supporting information

S1 File (DOCX)

S2 File (DOCX)

S3 File (DOCX)

S4 File (XLSX)

S5 File (DOCX)
