# Supplementary material for: Patient experiences of treatment-resistant depression (TRD): A systematic review and qualitative meta-synthesis
Source: PLOS Ment Health. 2024 Nov 4;1(6):e0000128. doi: 10.1371/journal.pmen.0000128 (PMC12798643; doi:10.1371/journal.pmen.0000128)
Supplement: S2 File — (DOCX) [file pmen.0000128.s002.docx]

**SUPPLEMENTARY FILE 2**

**Table S1:** Medline search strategy. Search was executed on February 9, 2024.

| **Line #** | **Search Terms** | **Number of References** |
| --- | --- | --- |
| 1 | exp Depressive Disorder, Major/ | 39,774 |
| 2 | exp Depressive Disorder, Treatment-Resistant/ | 2,241 |
| 3 | "depressive disorder, major".ab,kw,ti,tw. | 113 |
| 4 | "major depress".ab,kw,ti,tw. | 60,588 |
| 5 | "depressive disorder, treatment-resistant".ab,kw,ti,tw. | 21 |
| 6 | "treatment resistant depress*".ab,kw,ti,tw. | 4,140 |
| 7 | "refractory depress*".ab,kw,ti,tw. | 741 |
| 8 | "depress*".ab,kw,ti,tw. | 583,947 |
| **9** | **or/1-8** | **587,956** |
| 10 | exp Depressive Disorder, Treatment-Resistant/ | 2,241 |
| 11 | (depress* and ((antidepress* or SSRI* or SNRI* or (serotonin adj3 ("uptake" or "reuptake" or "re‐uptake")) or medication* or psychotropic or treatment* or respon*) adj2 fail*)).ti,ab,kf. | 1,993 |
| 12 | (depress* and ((antidepress* or SSRI* or SNRI* or (serotonin adj3 ("uptake" or "reuptake" or "re‐uptake")) or psychotropic medication* or treatment*) adj2 ("no response" or "not responsive" or "not responding" or "nonresponsive" or "non‐responsive" or unresponsive))).ti,ab,kf. | 134 |
| 13 | (depress* adj3 (refractor* or resistan* or chronic* or persist*)).ti,ab,kf. | 16,508 |
| 14 | (depress* adj3 (relaps* or recurr*)).ti,kf. | 1,572 |
| 15 | (depress* and (augment* or potentiat*)).mp. | 18,647 |
| **16** | **or/10-15** | **37,307** |
| 17 | exp Attitude to Health/ | 474,779 |
| 18 | exp Health Knowledge, Attitudes, Practice/ | 127,819 |
| 19 | exp Patient Preference/ | 10,849 |
| 20 | "attitud*".ab,kw,ti,tw. | 203,490 |
| 21 | "attitude to health".ab,kw,ti,tw. | 935 |
| 22 | "health knowledge, attitudes, practice".ab,kw,ti,tw. | 183 |
| 23 | "health know*".ab,kw,ti,tw. | 5,114 |
| 24 | "perspective*".ab,kw,ti,tw. | 474,120 |
| 25 | "view*".ab,kw,ti,tw. | 570,210 |
| 26 | "opinion*".ab,kw,ti,tw. | 134,984 |
| 27 | "perception*".ab,kw,ti,tw. | 343,395 |
| 28 | "judgement*".ab,kw,ti,tw. | 19,335 |
| 29 | "preferen*".ab,kw,ti,tw. | 345,919 |
| 30 | "patient preference*".ab,kw,ti,tw. | 12,575 |
| 31 | "value*".ab,kw,ti,tw. | 2,411,584 |
| 32 | "social value*".ab,kw,ti,tw. | 2,405 |
| 33 | "belie*".ab,kw,ti,tw. | 364,335 |
| 34 | "health belie*".ab,kw,ti,tw. | 7,372 |
| 35 | "experienc*".ab,kw,ti,tw. | 1,471,457 |
| **36** | **or/17-35** | **5,774,493** |
| 37 | exp Antidepressive Agents/ | 162,260 |
| 38 | "antidepressive agent*".ab,kw,ti,tw. | 1,717 |
| 39 | "antidepress*".ab,kw,ti,tw. | 80,305 |
| 40 | (antidepress* adj3 switch*".ab,kw,ti,tw. | 406 |
| 41 | (augment* adj5 (depress* or antidepress*)).ab,kw,ti,tw. | 1,910 |
| **42** | **or/37-41** | **194,660** |
| 43 | Qualitative Research/ | 85,374 |
| 44 | Interview/ | 30,934 |
| 45 | (theme$ or thematic).mp. | 182,155 |
| 46 | qualitative.af. | 364,933 |
| 47 | Nursing Methodology Research/ | 16,409 |
| 48 | questionnaire$.mp. | 985,727 |
| 49 | ethnological research.mp. | 8 |
| 50 | ethnograph$.mp. | 14,524 |
| 51 | ethnonursing.af. | 130 |
| 52 | phenomenol$.af. | 35,899 |
| 53 | (grounded adj (theor$ or study or studies or research or analys?s)).af. | 16,215 |
| 54 | (life stor$ or women* stor$).mp. | 1,818 |
| 55 | (emic or etic or hermeneutic$ or heuristic$ or semiotic$).af. or (data adj1 saturat$).tw. or participant observ$.tw. | 31,995 |
| 56 | (social construct$ or (postmodern$ or post-structural$) or (post structural$ or poststructural$) or post modern$ or post-modern$ or feminis$ or interpret$).mp. | 643,920 |
| 57 | (action research or cooperative inquir$ or co operative inquir$ or co-operative inquir$).mp. | 5,988 |
| 58 | (humanistic or existential or experiential or paradigm$).mp. | 202,188 |
| 59 | (field adj (study or studies or research)).tw. | 20,236 |
| 60 | human science.tw. | 267 |
| 61 | biographical method.tw. | 23 |
| 62 | theoretical sampl$.af. | 967 |
| 63 | ((purpos$ adj4 sampl$) or (focus adj group$)).af. | 95,637 |
| 64 | (account or accounts or unstructured or openended or open ended or text$ or narrative$).mp. | 856,159 |
| 65 | (life world or life-world or conversation analys?s or personal experience$ or theoretical saturation).mp. | 18,389 |
| 66 | ((lived or life) adj experience$).mp. | 20,968 |
| 67 | cluster sampl$.mp. | 9,875 |
| 68 | observational method$.af. | 1,024 |
| 69 | content analysis.af. | 46,210 |
| 70 | (constant adj (comparative or comparison)).af. | 6,238 |
| 71 | ((discourse$ or discurs$) adj3 analys?s).tw. | 3,184 |
| 72 | narrative analys?s.af. | 2,026 |
| 73 | heidegger$.tw. | 794 |
| 74 | colaizzi$.tw. | 1,276 |
| 75 | spiegelberg$.tw. | 86 |
| 76 | (van adj manen$).tw. | 570 |
| 77 | (van adj kaam$).tw. | 45 |
| 78 | (merleau adj ponty$).tw | 271 |
| 79 | husserl$.tw. | 328 |
| 80 | foucault$.tw. | 980 |
| 81 | (corbin$ adj2 strauss$).tw. | 466 |
| 82 | glaser$.tw. | 1,108 |
| **83** | **or/43-82** | **3,026,365** |
| **84** | **((9 and 42) or 16) and 36 and 83** | **4,314** |
| **85** | **limit 84 to yr="1987-Current"** | **4,216** |

**Table S2:** CINAHL search strategy. Search was executed on February 9, 2024.

| **Line #** | **Search Terms** | **Number of References** |
| --- | --- | --- |
| S1 | (MH "Depression+") | 138,257 |
| S2 | depressive disorder, major | 29 |
| S3 | "major depress*" | 17,385 |
| S4 | depressive disorder, treatment-resistant | 4 |
| S5 | "treatment resistant depress*" | 1,295 |
| S6 | depress* | 230,581 |
| S7 | “refractory depress*” | 136 |
| **S8** | **S1 or S2 or S3 or S4 or S5 or S6 or S7** | **230,655** |
| S9 | (MH "Depression+") and ("treatment resistant" or TRD or chronic or non-remission or "treatment refractory") | 13,791 |
| S10 | depress* N3 treatment-resistant | 1,476 |
| S11 | (depress* and ((antidepress* or SSRI* or SNRI* or (serotonin N3 ("uptake" or "reuptake" or "re‐uptake")) or medication* or psychotropic or treatment* or respon*) N2 fail*)) | 1,048 |
| S12 | (depress* and ((antidepress* or SSRI* or SNRI* or (serotonin N3 ("uptake" or "reuptake" or "re‐uptake")) or psychotropic medication* or treatment*) N2 ("no response" or "not responsive" or "not responding" or "nonresponsive" or "non‐responsive" or unresponsive))) | 203 |
| S13 | (depress* N3 (refractor* or resistan* or chronic* or persist*)) | 6,540 |
| S14 | (depress* N3 (relaps* or recurr*)) | 1,744 |
| S15 | (depress* and (augment* or potentiat*)) | 2,104 |
| **S16** | **S9 or S10 or S11 or S12 or S13 or S14 or S15** | **20,814** |
| S17 | (MH "Attitude to Health+") | 182,667 |
| S18 | (MH "Health Knowledge") | 38,837 |
| S19 | (MH "Patient Preference") | 2,969 |
| S20 | attitud* | 417,440 |
| S21 | attitude to health | 49,592 |
| S22 | health knowledge, attitudes, practice | 10 |
| S23 | health know* | 40,362 |
| S24 | perspective* | 176,376 |
| S25 | view* | 155,888 |
| S26 | opinion* | 52,260 |
| S27 | "perception*" | 195,908 |
| S28 | judgement* | 6,979 |
| S29 | preferen* | 66,598 |
| S30 | patient preference* | 9,082 |
| S31 | value* | 586,245 |
| S32 | social value* | 7,279 |
| S33 | belie* | 116,255 |
| S34 | (MH "Health Beliefs") | 16,185 |
| S35 | health belie* | 20,113 |
| S36 | experienc* | 602,485 |
| **S37** | **S17 or S18 or S19 or S20 or S21 or S22 or S23 or S24 or S25 or S26 or S27 or S28 or S29 or S30 or S31 or S32 or S33 or S34 or S35 or S36** | **1,861,899** |
| S38 | (MH "Antidepressive Agents+") | 25,758 |
| S39 | antidepressive agent* | 18,006 |
| S40 | antidepress* | 26,996 |
| S41 | antidepress* N3 switch* | 171 |
| S42 | augment* N5 (depress* or antidepress*) | 504 |
| **S43** | **S38 or S39 or S40 or S41 or S42** | **33,969** |
| S44 | MH Interview+ | 0 |
| S45 | MH audiorecording | 48,178 |
| S46 | MH Interviews+ | 262,668 |
| S47 | MH Grounded theory | 18,529 |
| S48 | MH Qualitative Studies | 151,401 |
| S49 | MH Research, Nursing | 22,845 |
| S50 | MH Questionnaires+ | 500,358 |
| S51 | MH Focus Groups | 51,938 |
| S52 | MH Discourse Analysis | 5,663 |
| S53 | MH Content Analysis | 45,584 |
| S54 | MH Ethnographic Research | 9,371 |
| S55 | MH Ethnological Research | 6,965 |
| S56 | MH Ethnonursing Research | 222 |
| S57 | MH Constant Comparative Method | 7,542 |
| S58 | MH Qualitative Validity+ | 1,876 |
| S59 | MH Purposive Sample | 40,871 |
| S60 | MH Observational Methods+ | 22,044 |
| S61 | MH Field Studies | 3,684 |
| S62 | MH theoretical sample | 1,975 |
| S63 | MH Phenomenology | 4,342 |
| S64 | MH Phenomenological Research | 19,962 |
| S65 | MH Life Experiences+ | 63,038 |
| S66 | MH Cluster Sample+ | 6,513 |
| S67 | Ethnonursing | 314 |
| S68 | ethnograph* | 15,290 |
| S69 | phenomenol* | 30,061 |
| S70 | grounded N1 theor* | 22,381 |
| S71 | grounded N1 study | 2,604 |
| S72 | grounded N1 studies | 2,604 |
| S73 | grounded N1 research | 501 |
| S74 | grounded N1 analys?s | 850 |
| S75 | life stor* | 1,440 |
| S76 | women’s stor* | 266 |
| S77 | emic or etic or hermeneutic$ or heuristic$ or semiotic$ | 7,845 |
| S78 | data N1 saturat* | 1,461 |
| S79 | participant observ* | 9,145 |
| S80 | social construct* or postmodern* or post-structural* or post structural* or poststructural* or post modern* or post-modern* or feminis* or interpret* | 132,084 |
| S81 | action research or cooperative inquir* or co operative inquir* or co-operative inquir* | 9,919 |
| S82 | humanistic or existential or experiential or paradigm* | 52,228 |
| S83 | field N1 stud* | 7,711 |
| S84 | field N1 research | 3,481 |
| S85 | human science | 377 |
| S86 | biographical method | 28 |
| S87 | theoretical sampl* | 2,465 |
| S88 | purpos* N4 sampl* | 47,055 |
| S89 | focus N1 group* | 64,590 |
| S90 | account or accounts or unstructured or open-ended or open ended or text* or narrative* | 227,500 |
| S91 | life world or life-world or conversation analys?s or personal experience* or theoretical saturation | 7,668 |
| S92 | lived experience* | 11,945 |
| S93 | life experience* | 44,021 |
| S94 | cluster sampl* | 7,576 |
| S95 | theme* or thematic | 158,768 |
| S96 | observational method* | 14,768 |
| S97 | questionnaire* | 622,231 |
| S98 | content analysis | 55,911 |
| S99 | discourse* N3 analys?s | 6,688 |
| S100 | discurs* N3 analys?s | 417 |
| S101 | constant N1 comparative | 9,448 |
| S102 | constant N1 comparison | 1,771 |
| S103 | narrative analysis | 1,672 |
| S104 | Heidegger* | 1,142 |
| S105 | Colaizzi* | 1,476 |
| S106 | Spiegelberg* | 91 |
| S107 | van N1 manen* | 936 |
| S108 | van N1 kaam* | 204 |
| S109 | "merleau N1 ponty*" | 0 |
| S110 | husserl* | 302 |
| S111 | Foucault* | 910 |
| S112 | Corbin* N2 strauss* | 507 |
| S113 | strauss* N2 corbin* | 507 |
| S114 | glaser* | 2,282 |
| **S115** | **S44 or S45 or S46 or S47 or S48 or S49 or S50 or S51 or S52 or S53 or S54 or S55 or S56 or S57 or S58 or S59 or S60 or S61 or S62 or S63 or S64 or S65 or S66 or S67 or S68 or S69 or S70 or S71 or S72 or S73 or S74 or S75 or S76 or S77 or S78 or S79 or S80 or S81 or S82 or S83 or S84 or S85 or S86 or S87 or S88 or S89 or S90 or S91 or S92 or S93 or S94 or S95 or S96 or S97 or S98 or S99 or S100 or S101 or S102 or S103 or S104 or S105 or S106 or S107 or S108 or S109 or S110 or S111 or S112 or S113 or S114** | **1,314,431** |
| **S116** | **((S8 and S43) or S16) and S37 and S115 (limited to a publication date of 1987-01-01 to present)** | **4,527** |

**Table S3:** PsycINFO search strategy. Search was executed on February 9, 2024.

| **Line #** | **Search Terms** | **Number of References** |
| --- | --- | --- |
| 1 | exp Major Depression/ | 165,856 |
| 2 | exp Treatment Resistant Depression/ | 3,186 |
| 3 | "depressive disorder, major".mp. | 19,946 |
| 4 | "major depress*".mp. | 169,600 |
| 5 | "depressive disorder, treatment-resistant".mp. | 727 |
| 6 | "treatment resistant depress*".mp. | 4,203 |
| 7 | "refractory depress*".mp. | 686 |
| 8 | depress*.mp. | 443,699 |
| **9** | **or/1-8** | **443,878** |
| 10 | exp Treatment Resistant Depression/ | 3,186 |
| 11 | (depress* and ((antidepress* or SSRI* or SNRI* or (serotonin adj3 ("uptake" or "reuptake" or "re‐uptake")) or medication* or psychotropic or treatment* or respon*) adj2 fail*)).mp. | 1,815 |
| 12 | (depress* and ((antidepress* or SSRI* or SNRI* or (serotonin adj3 ("uptake" or "reuptake" or "re‐uptake")) or psychotropic medication* or treatment*) adj2 ("no response" or "not responsive" or "not responding" or "nonresponsive" or "non‐responsive" or unresponsive))).mp. | 112 |
| 13 | (depress* adj3 (refractor* or resistan* or chronic* or persist*)).mp. | 12,828 |
| 14 | (depress* adj3 (relaps* or recurr*)).mp. | 5,666 |
| 15 | (depress* and (augment* or potentiat*)).mp. | 8,521 |
| **16** | **or/10-15** | **26,476** |
| 17 | exp Health Attitudes/ | 21,041 |
| 18 | exp Health Care Seeking Behavior/ | 10,361 |
| 19 | exp Health Knowledge/ | 9,592 |
| 20 | exp Preferences/ | 36,559 |
| 21 | exp Client Attitudes/ | 26,059 |
| 22 | exp Client Characteristics/ | 52,611 |
| 23 | exp Client Participation/ | 3,308 |
| 24 | exp Choice Behavior/ | 58,824 |
| 25 | exp Decision Making/ | 148,355 |
| 26 | "health attitude*".mp. | 12,673 |
| 27 | "health care seeking behavio*".mp. | 6,404 |
| 28 | "health knowledge".mp. | 36,178 |
| 29 | "preferen*".mp. | 146,138 |
| 30 | "client attitude*".mp. | 20,197 |
| 31 | "client characteristic*".mp. | 19,187 |
| 32 | "client participat*".mp. | 3,512 |
| 33 | "choice behavio*".mp. | 34,551 |
| 34 | "decision mak*".mp. | 159,618 |
| 35 | "attitude*".mp. | 557,726 |
| 36 | "attitude to health".mp. | 25,709 |
| 37 | “health knowledge, attitudes, practice".mp. | 28,318 |
| 38 | "health know*".mp. | 36,220 |
| 39 | "perspective*".mp. | 348,110 |
| 40 | "view*".mp. | 350,720 |
| 41 | "opinion*".mp. | 66,029 |
| 42 | "perception*".mp. | 612,946 |
| 43 | "judgement*".mp. | 12,932 |
| 44 | "preferen*".mp. | 146,138 |
| 45 | "patient preferen*".mp. | 3,814 |
| 46 | "value*".mp. | 404,641 |
| 47 | exp Social Values/ | 5,458 |
| 48 | exp Adult Attitudes/ | 13,010 |
| 49 | "social value*".mp. | 13,295 |
| 50 | "adult attitud*".mp. | 13,213 |
| 51 | belie*".mp. | 254,374 |
| 52 | "health belie*".mp. | 5,340 |
| 53 | "experienc*".mp. | 869,973 |
| **54** | **or/17-53** | **2,583,959** |
| 55 | exp Antidepressant Drugs/ | 42,141 |
| 56 | exp Drug Augmentation/ | 1,331 |
| 57 | exp Drug Therapy/ | 189,175 |
| 58 | "antidepressant drug*".mp. | 24,499 |
| 59 | "drug augment*".mp. | 1,358 |
| 60 | "drug therap*".mp. | 155,953 |
| 61 | "antidepressive agent*".mp. | 21,165 |
| 62 | "antidepress*".mp. | 54,299 |
| 63 | (antidepressant* adj3 switch*).mp. | 340 |
| 64 | (augment* adj5 (depress* or antidepress*)).mp. | 1,356 |
| **65** | **or/55-64** | **223,403** |
| 66 | exp Qualitative Methods/ | 21,815 |
| 67 | exp Interviews/ | 19,903 |
| 68 | (theme$ or thematic).mp. | 181,694 |
| 69 | qualitative.af. | 584,642 |
| 70 | "nursing methodology research".mp. | 4,990 |
| 71 | questionnaire$.mp. | 567,600 |
| 72 | "ethnological research".mp. | 14 |
| 73 | ethnograph$.mp. | 35,520 |
| 74 | ethnonursing.af | 315 |
| 75 | phenomenol$.af. | 163,046 |
| 76 | (grounded adj (theor$ or study or studies or research or analys?s)).af. | 82,191 |
| 77 | (life stor$ or women* stor$).mp. | 4,933 |
| 78 | (emic or etic or hermeneutic$ or semiotic$).af. or (data adj1 saturat$).tw. or participant observ$.tw. | 62,419 |
| 79 | (social construct$ or (postmodern$ or post-structural$) or (post structural$ or poststructural$) or post modern$ or post-modern$ or feminis$ or interpret$).mp. | 287,305 |
| 80 | (action research or cooperative inquir$ or co operative inquir$ or co-operative inquir$).mp. | 12,454 |
| 81 | (humanistic or existential or experiential or paradigm$).mp. | 160,995 |
| 82 | (field adj (study or studies or research)).tw. | 11,902 |
| 83 | human science.tw. | 692 |
| 84 | biographical method.tw. | 66 |
| 85 | theoretical sampl$.af. | 1,396 |
| 86 | ((purpos$ adj4 sampl$) or (focus adj group$)).af. | 97,525 |
| 87 | (account or accounts or unstructured or openended or open ended or text$ or narrative$).mp. | 424,157 |
| 88 | (life world or life-world or conversation analys?s or personal experience$ or theoretical saturation).mp. | 18,267 |
| 89 | ((lived or life) adj experience$).mp. | 59,816 |
| 90 | cluster sampl$.mp. | 2,158 |
| 91 | observational method$.af. | 3,222 |
| 92 | content analysis.af. | 90,055 |
| 93 | (constant adj (comparative or comparison)).af. | 7,725 |
| 94 | ((discourse$ or discurs$) adj3 analys?s).tw. | 11,326 |
| 95 | narrative analys?s.af. | 11,611 |
| 96 | heidegger$.tw. | 2,114 |
| 97 | colaizzi$.tw. | 783 |
| 98 | spiegelberg$.tw. | 19 |
| 99 | (van adj manen$).tw. | 666 |
| 100 | (van adj kaam$).tw. | 556 |
| 101 | (merleau adj ponty$).tw. | 1,077 |
| 102 | husserl$.tw. | 1,471 |
| 103 | foucault$.tw. | 3,410 |
| 104 | (corbin$ adj2 strauss$).tw. | 788 |
| 105 | glaser$.tw. | 1,347 |
| **106** | **or/66-105** | **1,923,906** |
| **107** | **((9 and 65) or 16) and 54 and 106** | **8,108** |
| **108** | **limit 107 to yr="1987 -Current"** | **7,925** |

**Table S4:** Web of Science Core Collection search strategy. Search was executed on February 9, 2024.

| **Line #** | **Search Terms** | **Number of References** |
| --- | --- | --- |
| 1 | TS=depressive disorder, major | 61,520 |
| 2 | TS=major depress* | 133,826 |
| 3 | TS=depressive disorder, treatment-resistant | 3,467 |
| 4 | TS=treatment resistant depress* | 11,835 |
| 5 | TS=depress* | 811,161 |
| 6 | TS=refractory depress* | 4,703 |
| **7** | **1 or 2 or 3 or 4 or 5 or 6** | **811,161** |
| 8 | TS=depressive disorder, treatment-resistant | 3,467 |
| 9 | TS=(depress* and ((antidepress* or SSRI* or SNRI* or (serotonin NEAR/3 ("uptake" or "reuptake" or "re‐uptake")) or medication* or psychotropic or treatment* or respon*) NEAR/2 fail*)) | 2,583 |
| 10 | TS=(depress* and ((antidepress* or SSRI* or SNRI* or (serotonin NEAR/3 ("uptake" or "reuptake" or "re‐uptake")) or "psychotropic medication*" or treatment*) NEAR/2 ("no response" or "not responsive" or "not responding" or "nonresponsive" or "non‐responsive" or unresponsive))) | 308 |
| 11 | TS=(depress* NEAR/3 (refractor* or resistan* or chronic* or persist*)) | 23,464 |
| 12 | TS=(depress* NEAR/3 (relaps* or recurr*)) | 7,110 |
| 13 | TS=(depress* and (augment* or potentiat*)) | 22,134 |
| **14** | **8 or 9 or 10 or 11 or 12 or 13** | **52,001** |
| 15 | TS=attitud* | 523,283 |
| 16 | TS=attitude to health | 120,609 |
| 17 | TS=health knowledge, attitudes, practice | 18,920 |
| 18 | TS=health know* | 464,382 |
| 19 | TS=perspective* | 1,324,352 |
| 20 | TS=view* | 1,425,990 |
| 21 | TS=opinion* | 242,770 |
| 22 | TS=perception* | 850,685 |
| 23 | TS=judgement* | 37,817 |
| 24 | TS=preferen* | 721,842 |
| 25 | TS=patient preference* | 62,174 |
| 26 | TS=value* | 5,507,072 |
| 27 | TS=social value* | 215,988 |
| 28 | TS=belie* | 678,084 |
| 29 | TS=health belie* | 96,453 |
| 30 | TS=experienc* | 2,467,692 |
| **31** | **15 or 16 or 17 or 18 or 19 or 20 or 21 or 22 or 23 or 24 or 25 or 26 or 27 or 28 or 29 or 30** | **11,942,450** |
| 32 | TS=antidepressive agent* | 1,142 |
| 33 | TS=antidepress* | 92,847 |
| 34 | TS=(antidepress* NEAR/3 switch*) | 464 |
| 35 | TS=(augment* NEAR/5 depress*) | 1,282 |
| 36 | TS=(augment* NEAR/5 antidepress*) | 968 |
| **37** | **32 or 33 or 34 or 35 or 36** | **93,634** |
| 38 | TS=interview* | 752,654 |
| 39 | TS=theme* | 267,951 |
| 40 | TS=thematic analysis | 88,237 |
| 41 | TS=qualitative | 696,269 |
| 42 | TS=nursing research methodology | 5,388 |
| 43 | TS=questionnaire | 879,807 |
| 44 | TS=ethnograph* | 78,758 |
| 45 | TS=ethnonursing | 78 |
| 46 | TS=ethnological research | 421 |
| 47 | TS=phenomenol* | 135,962 |
| 48 | (TS=grounded theor*) or (TS=grounded stud*) or (TS=grounded research) or (TS=grounded analys?s) | 592,788 |
| 49 | (TS=life stor*) or (TS=women’s stor*) | 136,393 |
| 50 | (TS=emic) or (TS=etic) or (TS=hermeneutic) or (TS=heuristic) or (TS=semiotic) or (TS=data saturat*) or (TS=participant observ*) | 541,661 |
| 51 | (TS=social construct*) or (TS=postmodern*) or (TS=post structural*) or (TS=feminis*) or (TS=interpret*) | 1,499,521 |
| 52 | (TS=action research) or (TS=co-operative inquir*) | 204,304 |
| 53 | (TS=humanistic) or (TS=existential) or (TS=experiential) or (TS=paradigm*) | 406,738 |
| 54 | (TS=field stud*) or (TS=field research) | 2,151,032 |
| 55 | TS=human science | 178,185 |
| 56 | TS=biographical method* | 2,910 |
| 57 | TS=theoretical sampl* | 116,838 |
| 58 | TS=purposive sampl* | 22,618 |
| 59 | (TS=open-ended account*) or (TS=unstructured account) or (TS=narrative*) or (TS=text*) | 1,140,118 |
| 60 | (TS=life world) or (TS=conversation analys?s) or (TS=theoretical saturation) | 180,945 |
| 61 | (TS=lived experience*) or (TS=life experience*) | 401,564 |
| 62 | TS=cluster sampl* | 170,823 |
| 63 | TS=observational method* | 201,173 |
| 64 | TS=content analysis | 719,609 |
| 65 | TS=constant comparative | 20,682 |
| 66 | (TS=discourse analys?s) or (TS=discurs* analys?s) | 84,946 |
| 67 | TS=narrative analys?s | 64,094 |
| 68 | TS=heidegger* | 12,239 |
| 69 | TS=colaizzi* | 1,218 |
| 70 | TS=spiegelberg* | 118 |
| 71 | TS=van manen* | 620 |
| 72 | TS=van kaam* | 49 |
| 73 | TS=merleau ponty* | 2,779 |
| 74 | TS=husserl* | 6,156 |
| 75 | TS=foucault* | 14,035 |
| 76 | TS=corbin* | 1,825 |
| 77 | TS=strauss* | 15,163 |
| 78 | TS=glaser* | 2,705 |
| **79** | **38 or 39 or 40 or 41 or 42 or 43 or 44 or 45 or 46 or 47 or 48 or 49 or 50 or 51 or 52 or 53 or 54 or 55 or 56 or 57 or 58 or 59 or 60 or 61 or 62 or 63 or 64 or 65 or 66 or 67 or 68 or 69 or 70 or 71 or 72 or 73 or 74 or 75 or 76 or 77 or 78** | **9,277,856** |
| **80** | **((7 and 37) or 14) and 31 and 79** | **8,575** |
