# Supplementary material for: Patient experiences of treatment-resistant depression (TRD): A systematic review and qualitative meta-synthesis
Source: PLOS Ment Health. 2024 Nov 4;1(6):e0000128. doi: 10.1371/journal.pmen.0000128 (PMC12798643; doi:10.1371/journal.pmen.0000128)
Supplement: S3 File — (DOCX) [file pmen.0000128.s003.docx]

**SUPPLEMENTARY FILE 3**

**Table S5:** Articles proceeded to full text review, whether they were included or excluded, and the reasons for exclusion (if applicable).

| **#** | **First Author (Year)** | **Title** | **Source of Reference** | **Included?** | **Reason for Exclusion** |
| --- | --- | --- | --- | --- | --- |
| 1 | **Adams (2006)** | Differential effectiveness of depression disease management for rural and urban primary care patients | electronic search | no | quantitative study |
| 2 | **Allen (2009)** | Participants' experiences of mindfulness-based cognitive therapy: "It changed me in just about every way possible." | electronic search | no | wrong population (study participants have depression but not TRD or unclear if TRD) |
| 3 | **Anderson (2013)** | Patient experiences of taking antidepressants for depression: a secondary qualitative analysis | electronic search | no | wrong population (study participants have depression but not TRD or unclear if TRD) |
| 4 | **Aselton (2010)** | The lived experience of college students who have been medicated with antidepressants | electronic search | no | thesis or pre-print |
| 5 | **Badger (2007)** | Use of self-chosen therapies by depressed people in primary care | electronic search | no | wrong population (study participants have depression but not TRD or unclear if TRD) |
| 6 | **Badger (2007)** | Attributing recovery from depression. Perceptions of people cared for in primary care | electronic search | no | wrong population (study participants have depression but not TRD or unclear if TRD) |
| 7 | **Baune (2021)** | Patient expectations and experiences of antidepressant therapy for major depressive disorder: a qualitative study | electronic search | no | wrong population (study participants have depression but not TRD or unclear if TRD) |
| 8 | **Bayliss (2015)** | Experiences of antidepressant medication and cognitive-behavioural therapy for depression: a grounded theory study | electronic search | no | wrong population (study participants have depression but not TRD or unclear if TRD) |
| 9 | **Bihari (2014)** | Relating mindfully: a qualitative exploration of changes in relationships through mindfulness-based cognitive therapy | electronic search | no | wrong population (study participants have depression but not TRD or unclear if TRD) |
| 10 | **Bogner (2009)** | Older primary care patient views regarding antidepressants: a mixed methods approach | electronic search | no | wrong population (study participants have depression but not TRD or unclear if TRD) |
| 11 | **Bosman (2016)** | Long-tern antidepressant use: a qualitative study on perspectives of patients and GPs in primary care | electronic search | no | wrong population (study participants have depression but not TRD or unclear if TRD) |
| 12 | **Breeksema (2022)** | Holding on or letting go? Patient experiences of control, context, and care in oral esketamine treatment for treatment-resistant depression: a qualitative study | electronic search | yes | n/a |
| 13 | **Breeksema (2023)** | Phenomenology and therapeutic potential of patient experiences during oral esketamine treatment for treatment-resistant depression: an interpretive phenomenological study | electronic search | yes | n/a |
| 14 | **Breeksema (2024)** | Patient perspectives and experiences with psilocybin treatment for treatment-resistant depression: a qualitative study | electronic search | yes | n/a |
| 15 | **Brijnath (2017)** | Playing with antidepressants: perspectives from Indian Australians and Anglo-Australians living with depression | electronic search | no | wrong population (study participants have depression but not TRD or unclear if TRD) |
| 16 | **Brown (2005)** | Beliefs about antidepressant medications in primary care patients: relationship to self-reported adherence | electronic search | no | quantitative study |
| 17 | **Brown (2008)** | Exploring perceptions of alcohol use as self-medication for depression among women receiving community-based treatment for alcohol problems | electronic search | no | wrong population (study participants have depression but not TRD or unclear if TRD) |
| 18 | **Brydges (2019)** | Men's views of antidepressant treatment for depression, and their implications for community pharmacy practice | electronic search | no | wrong population (study participants have depression but not TRD or unclear if TRD) |
| 19 | **Bull (2002)** | Discontinuation of use and switching of antidepressants: influence of patient-physician communication | electronic search | no | quantitative study |
| 20 | **Burrage (2016)** | Conditional beliefs of primary care patients with treatment-resistant depression | electronic search | no | wrong population (study participants have depression but not TRD or unclear if TRD) |
| 21 | **Buus (2014)** | Adherence to anti-depressant medication: a medicine-taking career | electronic search | no | wrong population (study participants have depression but not TRD or unclear if TRD) |
| 22 | **Buus (2012)** | Explanatory models of depression and treatment adherence to antidepressant medication: a qualitative interview study | electronic search | no | wrong population (study participants have depression but not TRD or unclear if TRD) |
| 23 | **Byrne (2020)** | Cohort profile: the Australian genetics of depression study | electronic search | no | did not focus on patient experiences or perspectives |
| 24 | **Cabassa (2007)** | Latino immigrant men's perceptions of depression and attitudes toward help seeking | electronic search | no | wrong population (study participants have depression but not TRD or unclear if TRD) |
| 25 | **Cabassa (2007)** | It's like being in a labyrinth:' Hispanic immigrants' perceptions of depression and attitudes toward treatments | electronic search | no | wrong population (study participants have depression but not TRD or unclear if TRD) |
| 26 | **Cabrera (2018)** | Interpreting patients' beliefs about deep brain stimulation for treatment-resistant depression: the need for caution and for context | electronic search | no | editorial, opinion or perspective piece, or commentary |
| 27 | **Camacho (2018)** | Descriptions of depression and depression treatment in older Hispanic immigrants in a geriatric collaborative care program | electronic search | no | wrong population (study participants have depression but not TRD or unclear if TRD) |
| 28 | **Cartwright (2018)** | Personal agency in women's recovery from depression: the impact of antidepressants and women's personal efforts | electronic search | no | wrong population (study participants have depression but not TRD or unclear if TRD) |
| 29 | **Castaneda (2020)** | User perspectives on cannabis and SSRIs as treatment for depression | electronic search | no | wrong population (study participants have depression but not TRD or unclear if TRD) |
| 30 | **Chalder (2012)** | A pragmatic randomised controlled trial to evaluate the cost-effectiveness of a physical activity intervention as a treatment for depression: the treating depression with physical activity (TREAD) trial | electronic search | no | wrong population (study participants have depression but not TRD or unclear if TRD) |
| 31 | **Chambers (2015)** | The self-management of longer-term depression: learning from the patient, a qualitative study | footnote chasing | no | wrong population (study participants have depression but not TRD or unclear if TRD) |
| 32 | **Chen (2022)** | Experiences and coping strategies of somatic symptoms in patients with depressive disorder: a qualitative study | electronic search | no | wrong population (study participants have depression but not TRD or unclear if TRD) |
| 33 | **Chew-Graham (2018)** | The value of an embedded qualitative study in a trial of a second antidepressant for people who had not responded to one antidepressant: understanding the perspectives of patients and general practitioners | electronic search | no | wrong population (study participants have depression but not TRD or unclear if TRD) |
| 34 | **Clarke (2018)** | Patients with depression who self-refer for transcranial magnetic stimulation for treatment: exploratory qualitative study | electronic search | no | did not focus on patient experiences or perspectives |
| 35 | **Cornish (2017)** | "They have said that I was slightly depressed but there are circumstances that bring that on": how middle-aged and older African American men describe perceived stress and depression | electronic search | no | wrong population (study participants have depression but not TRD or unclear if TRD) |
| 36 | **Crowe (2020)** | Patients' experiences of cognitive functioning in recurrent depression: a qualitative study | electronic search | no | wrong population (study participants have depression but not TRD or unclear if TRD) |
| 37 | **Dahlberg (2008)** | Mental health literacy and attitudes in a Swedish community sample - investigating the role of personal experience of mental health care | electronic search | no | wrong population (study did not focus specifically on depression) |
| 38 | **De Anta (2022)** | Areas of interest and social consideration of antidepressants on English tweets: a natural language processing classification study | electronic search | no | did not focus on patient experiences or perspectives |
| 39 | **Dickinson (2010)** | Long-term prescribing of antidepressants in the older population: a qualitative study | electronic search | no | wrong population (study participants have depression but not TRD or unclear if TRD) |
| 40 | **Dos Santos (2018)** | Long-term effects of ayahuasca in patients with recurrent depression: a 5-year qualitative follow-up | electronic search | no | wrong study setting (low- or middle-income country, or high-income country not part of the OECD or EEA) |
| 41 | **Ebert (2017)** | An ethnographic study of the effects of cognitive symptoms in patients with major depressive disorder: the IMPACT study | electronic search | no | wrong population (study participants have depression but not TRD or unclear if TRD) |
| 42 | **Erdal (2011)** | Attitudes about depression and its treatment among mental health professionals, lay persons, and immigrants and refugees in Norway | electronic search | no | quantitative study |
| 43 | **Fosgerau (2014)** | Patients' perspectives on antidepressant treatment in consultations with physicians | electronic search | no | wrong population (study participants have depression but not TRD or unclear if TRD) |
| 44 | **French (2017)** | Individuals' long term use of cognitive behavioural skills to manage their depression: a qualitative study | electronic search | no | wrong population (study participants have depression but not TRD or unclear if TRD) |
| 45 | **Fullager (2009)** | Negotiating the neurochemical self: anti-depressant consumption in women's recovery from depression | electronic search | no | wrong population (study participants have depression but not TRD or unclear if TRD) |
| 46 | **Gask (2011)** | Isolation, feeling 'stuck', and loss of control: understanding persistence of depression in British Pakistani women | electronic search | no | wrong population (study participants have depression but not TRD or unclear if TRD) |
| 47 | **Gebara (2020)** | Illness narratives and preferences for treatment among older veterans living with treatment-resistant depression and insomnia | electronic search | no | wrong population (study participants have depression but not TRD or unclear if TRD) |
| 48 | **Gilbert (2019)** | Legitimising depression: community perspectives and the help-seeking continuum | electronic search | no | wrong population (study participants have depression but not TRD or unclear if TRD) |
| 49 | **Green (2017)** | Knowledge and preferences regarding antidepressant medication among depressed Latino patients in primary care | electronic search | no | wrong population (study participants have depression but not TRD or unclear if TRD) |
| 50 | **Griffiths (2021)** | A qualitative study of patients' experience of ketamine treatment for depression: the 'Ketamine and Me' project | footnote chasing | yes | n/a |
| 51 | **Grime (2004)** | Information versus experience: a comparison of an information leaflet on antidepressants with lay experience of treatment | electronic search | no | wrong population (study participants have depression but not TRD or unclear if TRD) |
| 52 | **Guillaumie (2018)** | Patient perspectives on the role of community pharmacists for antidepressant treatment: a qualitative study | electronic search | no | wrong population (study participants have depression but not TRD or unclear if TRD) |
| 53 | **Hajela (2013)** | The monster within: understanding the narratives of depression | electronic search | no | wrong population (study participants have depression but not TRD or unclear if TRD) |
| 54 | **Hughes (2020)** | The micro-to-macro realities of antidepressant taking: users' experiences in the context of contested science and industry promotion | electronic search | no | wrong population (study participants have depression but not TRD or unclear if TRD) |
| 55 | **Huijbers (2020)** | Discontinuing antidepressant medication after mindfulness-based cognitive therapy: a mixed methods study exploring predictors and outcomes of different discontinuation trajectories, and its facilitators and barriers | electronic search | no | wrong population (study participants have depression but not TRD or unclear if TRD) |
| 56 | **Incecik (2019)** | True Colours monitoring in treatment resistant depression: a qualitative study of patients' perspectives | electronic search | no | unable to access full text |
| 57 | **Interian (2007)** | A qualitative analysis of the perception of stigma among Latinos receiving antidepressants | electronic search | no | wrong population (study participants have depression but not TRD or unclear if TRD) |
| 58 | **Interian (2011)** | The long-term trajectory of depression among Latinos in primary care and its relationship to depression care disparities | electronic search | no | wrong population (study participants have depression but not TRD or unclear if TRD) |
| 59 | **Izquierdo (2014)** | Older depressed Latinos' experiences with primary care visits for personal, emotional, and/or mental health problems: a qualitative analysis | electronic search | no | wrong population (study participants have depression but not TRD or unclear if TRD) |
| 60 | **Jaffray (2014)** | Why do patients discontinue antidepressant therapy early? A qualitative study | electronic search | no | wrong population (study participants have depression but not TRD or unclear if TRD) |
| 61 | **Jilka (2020)** | Ketamine treatment for depression: qualitative study exploring patient views | electronic search | no | wrong population (study participants have depression but not TRD or unclear if TRD) |
| 62 | **Johnson (2006)** | Influence of patient preference and primary care clinician proclivity for watchful waiting on receipt of depression treatment | electronic search | no | quantitative study |
| 63 | **Kaelen (2018)** | The hidden therapist: evidence for a central role of music in psychedelic therapy | electronic search | no | wrong topic |
| 64 | **Kaplan (2000)** | Childhood emotional trauma and chronic posttraumatic stress disorder in adult outpatients with treatment-resistant depression | electronic search | no | quantitative study |
| 65 | **Karasz (2012)** | What we talk about when we talk about depression: doctor-patient conversations and treatment decision outcomes | electronic search | no | wrong population (study participants have depression but not TRD or unclear if TRD) |
| 66 | **Kerr (2023)** | The lived experience of major and treatment-resistant depression in England: a mixed-methods study | electronic search | yes | n/a |
| 67 | **Kessing (2005)** | Depressive and bipolar disorders: patients' attitudes and beliefs towards depression and antidepressants | electronic search | no | quantitative study |
| 68 | **Kragh (2017)** | Experiences of wake and light therapy in depression: a qualitative study | electronic search | yes | n/a |
| 69 | **Kroch (2022)** | Order and disorder: navigating narrative tensions in the experience of treatment resistant depression | electronic search | yes | n/a |
| 70 | **Lapidos (2023)** | Patients’ recovery and non-recovery narratives after intravenous ketamine for treatment-resistant depression | electronic search | no | wrong population (paediatric population aged younger than 18 years, or adult population with excluded co-morbidities such as Alzheimer's disease, dementia, PTSD) |
| 71 | **Lascelles (2019)** | Effects of ketamine treatment on suicidal ideation: a qualitative study of patients' accounts following treatment for depression in a UK ketamine clinic | electronic search | yes | n/a |
| 72 | **Lascelles (2020)** | Ketamine treatment for individuals with treatment-resistant depression: longitudinal qualitative interview study of patient experiences | electronic search | yes | n/a |
| 73 | **Lauber (2003)** | Patients' attitudes toward antidepressants | electronic search | no | editorial, opinion or perspective piece, or commentary |
| 74 | **Lawrence (2006)** | Coping with depression in later life: a qualitative study of help-seeking in three ethnic groups | electronic search | no | wrong population (study participants have depression but not TRD or unclear if TRD) |
| 75 | **Lawrence (2018)** | Patients' beliefs about deep brain stimulation for treatment-resistant depression | electronic search | yes | n/a |
| 76 | **Leibold (2014)** | Activities and adaptation in late-life depression: a qualitative study | electronic search | no | wrong population (study participants have depression but not TRD or unclear if TRD) |
| 77 | **Lewis (2015)** | I'm managing myself': how and why people use St John's wort as a strategy to manage their mental health risk | electronic search | no | wrong population (study participants have depression but not TRD or unclear if TRD) |
| 78 | **Lewis (2019)** | Analysis of clinical trial exit data in patients with treatment-resistant depression | electronic search | no | quantitative study |
| 79 | **Leydon (2007)** | A qualitative study of patient views on discontinuing long-term selective seroronin reuptake inhibitors | electronic search | no | wrong population (study participants have depression but not TRD or unclear if TRD) |
| 80 | **Malpass (2010)** | Concordance between PHQ-9 scores and patients' experiences of depression: a mixed methods study | electronic search | no | wrong population (study participants have depression but not TRD or unclear if TRD) |
| 81 | **Malpass (2011)** | I didn't want her to panic: unvoiced patient agendas in primary care consultations when consulting about antidepressants | electronic search | no | wrong population (study participants have depression but not TRD or unclear if TRD) |
| 82 | **Maxwell (2005)** | Women's and doctors' accounts of their experiences of depression in primary care: the influence of social and moral reasoning on patients' and doctors' decisions | electronic search | no | wrong population (study participants have depression but not TRD or unclear if TRD) |
| 83 | **McCann (2012)** | Young people with depression and their satisfaction with the quality of care they receive from a primary care youth mental health service: a qualitative study | electronic search | no | wrong population (paediatric population aged younger than 18 years, or adult population with excluded co-morbidities such as Alzheimer's disease, dementia, PTSD) |
| 84 | **McGillivray (2010)** | Detecting and treating depression in people with mild intellectual disability: the views of key stakeholders | electronic search | no | unable to access full text |
| 85 | **McKeown (2022)** | Patient perspectives of lithium and quetiapine augmentation treatment in treatment-resistant depression: a qualitative assessment | electronic search | no | wrong population (paediatric population aged younger than 18 years, or adult population with excluded co-morbidities such as Alzheimer's disease, dementia, PTSD) |
| 86 | **McMullen (2009)** | Women's accounts of their decision to quit taking antidepressants | electronic search | no | wrong population (study participants have depression but not TRD or unclear if TRD) |
| 87 | **McNaughton (2019)** | Patient attitudes toward and goals for MDD treatment: a survey study | electronic search | no | wrong population (study participants have depression but not TRD or unclear if TRD) |
| 88 | **McPherson (2014)** | Treatment resistant depression in primary care: co-constructing difficult encounters | electronic search | no | did not focus on patient experiences or perspectives |
| 89 | **Mynatt (2005)** | Patients with anxiety and depression wanted to know what to expect when they started their medication | electronic search | no | editorial, opinion or perspective piece, or commentary |
| 90 | **Nicolaidis (2013)** | The Interconnections Project: development and evaluation of a community-based depression program for African American violence survivors | electronic search | no | wrong population (study participants have depression but not TRD or unclear if TRD) |
| 91 | **Nolan (2005)** | Aspects of the relationship between doctors and depressed patients that enhance satisfaction with primary care | electronic search | no | wrong population (study participants have depression but not TRD or unclear if TRD) |
| 92 | **Nygaard (2015)** | Balancing risk: a grounded theory study of pregnant women’s decisions to (dis)continue antidepressant therapy | electronic search | no | wrong population (study participants have depression but not TRD or unclear if TRD) |
| 93 | **O'Mullan (2014)** | Women's experiences of coping with the sexual side effects of antidepressant medication | electronic search | no | wrong population (study participants have depression but not TRD or unclear if TRD) |
| 94 | **Papageorgiou (2001)** | Metacognitive beliefs about rumination in recurrent major depression | electronic search | no | wrong population (study participants have depression but not TRD or unclear if TRD) |
| 95 | **Percival (2017)** | She believed in me'. What patients with depression value in their relationship with practitioners. A secondary analysis of multiple qualitative data sets | electronic search | no | wrong population (study participants have depression but not TRD or unclear if TRD) |
| 96 | **Pestello (2008)** | Taking anti-depressant medication: a qualitative examination of Internet postings | electronic search | no | unable to access full text |
| 97 | **Raffin Bouchal (2023)** | Personal recovery associated with deep brain stimulation for treatment-resistant depression: a constructivist grounded theory study | electronic search | yes | n/a |
| 98 | **Ragan (2010)** | Meaningful lives: elders in treatment for depression | electronic search | no | wrong population (study participants have depression but not TRD or unclear if TRD) |
| 99 | **Ridge (2015)** | "My dirty little habit": patient constructions of antidepressant use and the 'crisis' of legitimacy | electronic search | no | wrong population (study participants have depression but not TRD or unclear if TRD) |
| 100 | **Rhodes (2019)** | Emptiness, engulfment, and life struggle: an interpretive phenomenological analysis of chornic depression | electronic search | no | wrong population (study participants have depression but not TRD or unclear if TRD) |
| 101 | **Rodriguez-Galan (2014)** | Aging Puerto Ricans' experiences of depression treatment: a new ethnographic exploration | electronic search | no | wrong population (study participants have depression but not TRD or unclear if TRD) |
| 102 | **Rollman (2002)** | Race, quality of depression care, and recovery from major depression in a primary care setting | electronic search | no | quantitative study |
| 103 | **Rosedale (2009)** | The structure of the lived experience for persons having undergone rTMS for depression treatment | electronic search | no | protocol or paper reporting preliminary findings |
| 104 | **Schofield (2011)** | Patients' views of antidepressants: from first experiences to becoming expert | electronic search | no | wrong population (study participants have depression but not TRD or unclear if TRD) |
| 105 | **Schreiber (1996)** | (Re)defining my self: women's process of recovery from depression | electronic search | no | wrong population (study participants have depression but not TRD or unclear if TRD) |
| 106 | **Simmonds (2013)** | Patients' experiences of participating in a large-scale trial of cognitive behavioural therapy for depression: a mixed methods study | electronic search | no | did not focus on patient experiences or perspectives |
| 107 | **Sleath (2003)** | Drug information sources and antidepressant adherence | electronic search | no | wrong population (study participants have depression but not TRD or unclear if TRD) |
| 108 | **Starr (2020)** | Self-reported review of the value of esketamine in patients with treatment-resistant depression: understanding the patient experience in the STRIVE Study | electronic search | yes | n/a |
| 109 | **Sumner (2021)** | A qualitative and quantitative account of patients' experiences of ketamine and its antidepressant properties | electronic search | yes | n/a |
| 110 | **Tamburrino (2009)** | Antidepressant medication adherence: a study of primary care patients | electronic search | no | wrong population (study participants have depression but not TRD or unclear if TRD) |
| 111 | **Thomson (2018)** | How is a specialist depression service effective for persistent moderate to severe depressive disorder? A qualitative study of service user experience | electronic search | no | wrong population (study participants have depression but not TRD or unclear if TRD) |
| 112 | **Thomson (2021)** | "Nothing to lose, absolutely everything to gain": patient and caregiver expectations and subjective outcomes of deep brain stimulation for treatment-resistant depression | electronic search | yes | n/a |
| 113 | **Thomson (2023)** | Personal and relational changes following deep brain stimulation for treatment-resistant depression: a prospective qualitative study with patients and caregivers | electronic search | yes | n/a |
| 114 | **Tickell (2020)** | Recovery from recurrent depression with mindfulness-based cognitive therapy and antidepressants: a qualitative study with illustrative case studies | electronic search | no | wrong population (study participants have depression but not TRD or unclear if TRD) |
| 115 | **Uebelacker (2010)** | Open trial of Vinyasa yoga for persistently depressed individuals: evidence of feasibility and acceptability | electronic search | no | wrong population (study participants have depression but not TRD or unclear if TRD) |
| 116 | **Van Geffen (2011)** | The decision to continue or discontinue treatment: experiences and beliefs of users of selective serotonin-reuptake inhibitors in the initial months - a qualitative study | electronic search | no | wrong population (study participants have depression but not TRD or unclear if TRD) |
| 117 | **Van Schalkwyk (2018)** | Acute psychoactive effects of intravenous ketamine during treatment of mood disorders: analysis of the clinician administered dissociative state scale | electronic search | no | wrong population (study participants have depression but not TRD or unclear if TRD) |
| 118 | **van Grieken (2014)** | Patients’ perspectives on how treatment can impeded their recovery from depression | footnote chasing | no | wrong population (study participants have depression but not TRD or unclear if TRD) |
| 119 | **Van Tiem (2021)** | "A link to the outside:" patient perspectives on a mobile texting program to improve depression self-management | electronic search | no | wrong population (study participants have depression but not TRD or unclear if TRD) |
| 120 | **Vargas (2015)** | Toward a cultural adaptation of pharmacotherapy: Latino views of depression and antidepressant therapy | electronic search | no | wrong population (study participants have depression but not TRD or unclear if TRD) |
| 121 | **Verbeek-Heida (2006)** | Better safe than sorry - why patients prefer to stop using selective serotonin reuptake inhibitor (SSRI) antidepressants but are afraid to do so: results of qualitative study | electronic search | no | wrong population (study participants have depression but not TRD or unclear if TRD) |
| 122 | **Waite (2007)** | Exploring depression among a cohort of African American women | electronic search | no | wrong population (study participants have depression but not TRD or unclear if TRD) |
| 123 | **Waite (2009)** | Perspectives about depression: explanatory models among African-American women | electronic search | no | wrong population (study participants have depression but not TRD or unclear if TRD) |
| 124 | **Watts (2017)** | Patients' accounts of increased "connectedness" and "acceptance" after psilocybin for treatment-resistant depression | electronic search | yes | n/a |
| 125 | **Wentink (2019)** | Enhancing shared decision making about discontinuation of antidepressant medication: a concept-mapping study in primary and secondary mental health care | electronic search | no | wrong population (study participants have depression but not TRD or unclear if TRD) |
| 126 | **Wiles (2018)** | Management of treatment-resistant depression in primary care: a mixed methods study | electronic search | no | wrong population (study participants have depression but not TRD or unclear if TRD) |
| 127 | **Withers (2015)** | Perspectives of vulnerable US Hispanics with rheumatoid arthritis on depression: awareness, barriers to disclosure, and treatment options | electronic search | no | wrong population (study participants have depression but not TRD or unclear if TRD) |
| 128 | **Zolnoori (2018)** | Identifying the underlying factors associated with patients' attitudes toward antidepressants: qualitative and quantitative analysis of patient drug reviews | electronic search | no | wrong population (study participants have depression but not TRD or unclear if TRD) |
