# Supplementary material for: Patient experiences of treatment-resistant depression (TRD): A systematic review and qualitative meta-synthesis
Source: PLOS Ment Health. 2024 Nov 4;1(6):e0000128. doi: 10.1371/journal.pmen.0000128 (PMC12798643; doi:10.1371/journal.pmen.0000128)
Supplement: S5 File — (DOCX) [file pmen.0000128.s005.docx]

**SUPPLEMENTARY FILE 5**

Critical appraisal of studies included in the systematic literature review and qualitative meta-synthesis was performed using the Standards for Reporting Qualitative Research (SRQR) instrument [1]. The quality criteria and our judgement for each paper are presented in the tables below. We did not paraphrase the description of each criterion and instead provided it verbatim as it appears in the SRQR instrument [1]. We did not exclude any articles on the basis of this critical appraisal, in recognition of the fact that methodological and procedural details may often be omitted from a manuscript despite their presence in the research process.

**Table S5:** Critical appraisal of studies (part 1).

| **Criterion** | **Breeksema et al (2022) [2]** | **Breeksema et al (2023) [3]** | **Breeksema et al (2024) [4]** | **Griffiths et al (2021) [5]** | **Kerr et al (2023) [6]** | **Kragh et al (2017) [7]** |
| --- | --- | --- | --- | --- | --- | --- |
| **Title:** Concise description of the nature and topic of the study identifying the study as qualitative or indicating the approach (eg., ethnography, grounded theory) or data collection methods (eg., interview, focus group) is recommended. | The title identifies the study as qualitative but does not indicate the approach used. | The title clearly identifies the study as qualitative and indicates the approach used. | The title identifies the study as qualitative but does not indicate the approach used. | The title identifies the study as qualitative but does not indicate the approach used. | The title identifies the study as qualitative but does not indicate the approach used. | The title identifies the study as qualitative but does not indicate the approach used. |
| **Abstract:** Summary of key elements of the study using the abstract format of the intendent publication; typically includes background, purpose, methods, results, and conclusions. | All of the elements listed are present. | All of the elements listed are present. | Only some of the elements listed are present. | All of the elements listed are present. | All of the elements listed are present. | All of the elements listed are present. |
| **Problem formulation:** Description and significance of the problem/phenomenon studied; review of the relevant theory and empirical work; problem statement. | All of the elements listed are present. | All of the elements listed are present. | All of the elements listed are present. | Only some of the elements listed are present. | All of the elements listed are present. | All of the elements listed are present. |
| **Purpose or research question:** Purpose of the study and specific objectives or questions. | The purpose of the study or research question is not clearly stated. | The purpose of the study or research question is clearly stated. | The purpose of the study or research question is not clearly stated. | The purpose of the study or research question is clearly stated. | The purpose of the study or research question is not clearly stated. | The purpose of the study or research question is clearly stated. |
| **Qualitative approach and research paradigm:** Qualitative approach (eg., ethnography, grounded theory, case study, phenomenology, narrative research) and guiding theory if appropriate; identifying the research paradigm (eg., postpositivist, constructivist/ interpretivist) is also recommended; rationale. | The qualitative approach, research paradigm, and a rationale are all provided. | The qualitative approach, research paradigm, and a rationale are all provided. | The qualitative approach, research paradigm, and a rationale are all provided. | Only one of the qualitative approach (phenomenology) or the research paradigm are provided, and a rationale is included. | Neither a specific qualitative approach nor a research paradigm is provided. | Neither a specific qualitative approach nor a research paradigm is provided. |
| **Research characteristics and reflexivity:** Researchers’ characteristics that may influence the research, including personal attributes, qualifications/experience, relationship with participants, assumptions, and/or presuppositions; potential or actual interaction between researchers’ characteristics and the research questions, approach, methods, results, and/or transferability. | No mention of reflexivity is made. | No mention of reflexivity is made. | No mention of reflexivity is made. | No mention of reflexivity is made. | No mention of reflexivity is made. | No mention of reflexivity is made. |
| **Context:** Setting/site and salient contextual factors; rationale. | The study context and rationale are provided with sufficient detail. | The study context and rationale are provided but without adequate detail. | The study context and rationale are provided with sufficient detail. | The study context is provided but a rationale is not. | The study context and rationale are provided but without adequate detail. | The study context and rationale are provided with sufficient detail. |
| **Sampling strategy:** How and why research participants, documents, or events were selected; criteria for deciding when no further sampling was necessary (eg., sampling saturation); rationale. | The sampling strategy is described but a rationale is not provided. | The sampling strategy is described but a rationale is not provided. | The sampling strategy is described and a rationale is provided. | The sampling strategy is described but a rationale is not provided. | The sampling strategy is described but a rationale is not provided. | The sampling strategy is not described. |
| **Ethical issues pertaining to human subjects:** Documentation of approval by an appropriate ethics review board and participant consent, or explanation for lack thereof; other confidentiality and data security issues. | Documentation of ethics approval is provided (or an explanation for a lack thereof is given). | Documentation of ethics approval is provided (or an explanation for a lack thereof is given). | Documentation of ethics approval is provided (or an explanation for a lack thereof is given). | Documentation of ethics approval is provided (or an explanation for a lack thereof is given). | Documentation of ethics approval is provided (or an explanation for a lack thereof is given). | Documentation of ethics approval is provided (or an explanation for a lack thereof is given). |
| **Data collection methods:** Types of data collected; details of data collection procedures including (as appropriate) start and stop dates of data collection and analysis, iterative process, triangulation of sources/methods, and modification of procedures in response to evolving study findings; rationale. | Data collection methods are described but without adequate detail. | Data collection methods are described with sufficient detail. | Data collection methods are described but without adequate detail. | Data collection methods are described but without adequate detail. | Data collection methods are described with sufficient detail. | Data collection methods are described with sufficient detail. |
| **Data collection instruments and technologies:** Description of instruments (eg., interview guides, questionnaires) and devices (eg., audio recorders) used for data collection; if/how the instrument(s) changed over the course of the study. | Data collection instruments are described with sufficient detail. | Data collection instruments are described with sufficient detail. | Data collection instruments are described with sufficient detail. | Data collection instruments are described with sufficient detail. | Data collection instruments are described with sufficient detail. | Data collection instruments are described with sufficient detail. |
| **Units of study:** Number and relevant characteristics of participants, documents, or events included in the study; level of participation (could be reported in results). | Number and relevant characteristics of participants are provided but without sufficient detail. | Number and relevant characteristics of participants are provided with adequate detail. | Number and relevant characteristics of participants are provided but without sufficient detail. | Number and relevant characteristics of participants are provided but without sufficient detail. | Number and relevant characteristics of participants are provided but without sufficient detail. | Number and relevant characteristics of participants are provided but without sufficient detail. |
| **Data processing:** Methods for processing data prior to and during analysis, including transcription, data entry, data management and security, verification of data integrity, data coding, and anonymization/ deidentification of excerpts. | All of the elements listed are present. | All of the elements listed are present. | Only some of the elements listed are present. | Only some of the elements listed are present. | All of the elements listed are present. | Only some of the elements listed are present. |
| **Data analysis:** Process by which inferences, themes, etc., were identified and developed, including the researchers involved in data analysis; usually references a specific paradigm or approach; rationale. | Data analysis methods are described with sufficient detail. | Data analysis methods are described with sufficient detail. | Data analysis methods are described but without adequate detail. | Data analysis methods are described with sufficient detail. | Data analysis methods are described with sufficient detail. | Data analysis methods are described but without adequate detail. |
| **Techniques to enhance trustworthiness:** Techniques to enhance trustworthiness and credibility of data analysis (eg., member checking, audit trail, triangulation); rationale. | Techniques to enhance trustworthiness are described but without adequate detail. | Techniques to enhance trustworthiness are described with sufficient detail. | Techniques to enhance trustworthiness are not described. | Techniques to enhance trustworthiness are described with sufficient detail. | Techniques to enhance trustworthiness are described with sufficient detail. | Techniques to enhance trustworthiness are described but without adequate detail. |
| **Synthesis and interpretation:** Main findings (eg., interpretations, inferences, and themes); might include development of a theory or model, or integration with prior research or theory. | Main findings are described with sufficient detail. | Main findings are described with sufficient detail. | Main findings are described with sufficient detail. | Main findings are described with sufficient detail. | Main findings are described with sufficient detail. | Main findings are described with sufficient detail. |
| **Links to empirical data:** Evidence (eg., quotes, field notes, text excerpts, photographs) to substantiate analytic findings. | Links to empirical data are provided. | Links to empirical data are provided. | Links to empirical data are provided. | Links to empirical data are provided. | Links to empirical data are provided. | Links to empirical data are provided. |
| **Integration with prior work, implications, transferability, and contributions to the field:** Short summary of main findings; explanation of how findings and conclusions connect to, support, elaborate on, or challenge conclusions of earlier scholarship; discussion of scope of application/generalizability; identification of unique contribution(s) to scholarship in a discipline or field. | All of the elements listed are present. | All of the elements listed are present. | All of the elements listed are present. | Only some of the elements listed are present. Specifically, the findings of this study elaborate on previous findings, but it is not discussed whether this study makes a unique knowledge contribution. | All of the elements listed are present. | All of the elements listed are present. |
| **Limitations:** Trustworthiness and limitations of findings. | Limitations are listed and justification or mitigation strategies are provided. | Limitations are listed and justification or mitigation strategies are provided. | Limitations are listed and justification or mitigation strategies are provided. | Limitations are listed and justification or mitigation strategies are provided. | Limitations are listed but no justification or mitigation strategies are provided. | Limitations are listed but no justification or mitigation strategies are provided. |
| **Conflicts of interest:** Potential sources of influence or perceived influence on study conduct and conclusions; how these were managed. | Conflicts of interest are disclosed but no indication as to how these were managed is given. | Conflicts of interest are disclosed but no indication as to how these were managed is given. | Conflicts of interest are disclosed but no indication as to how these were managed is given. | A conflict of interest statement is provided and no conflicts of interest are disclosed. | Conflicts of interest are disclosed but no indication as to how these were managed is given. | A conflict of interest statement is not provided. |
| **Funding:** Sources of funding and other support; role of funders in data collection, interpretation, and reporting. | A funding statement is not provided. | A funding statement is not provided. | A funding statement is provided and no sources of funding support are listed. | A funding statement is provided and no sources of funding support are listed. | Sources of funding support are listed and the role of funders in data collection, interpretation, and/or reporting is provided. | A funding statement is not provided. |

**Table S7:** Critical appraisal of studies (part 2).

| **Criterion** | **Kroch et al (2021) [8]** | **Lascelles et al (2019) [9]** | **Lascelles et al (2020) [10]** | **Lawrence et al (2018) [11]** | **Raffin Bouchal et al (2023) [12]** | **Starr et al (2020) [13]** |
| --- | --- | --- | --- | --- | --- | --- |
| **Title:** Concise description of the nature and topic of the study identifying the study as qualitative or indicating the approach (eg., ethnography, grounded theory) or data collection methods (eg., interview, focus group) is recommended. | The title clearly identifies the study as qualitative and indicates the approach used. Although the word “qualitative” is not used in the title, we took the presence of “narrative” and “experience” to signal this was a qualitative paper. | The title identifies the study as qualitative but does not indicate the approach used. | The title identifies the study as qualitative but does not indicate the approach used. | The title neither identifies the study as qualitative nor indicates the approach used. | The title clearly identifies the study as qualitative and indicates the approach used. | The title neither identifies the study as qualitative nor indicates the approach used. |
| **Abstract:** Summary of key elements of the study using the abstract format of the intendent publication; typically includes background, purpose, methods, results, and conclusions. | All of the elements listed are present. | All of the elements listed are present. | All of the elements listed are present. | Only some of the elements listed are present. | All of the elements listed are present. | All of the elements listed are present. |
| **Problem formulation:** Description and significance of the problem/phenomenon studied; review of the relevant theory and empirical work; problem statement. | All of the elements listed are present. | All of the elements listed are present. | Only some of the elements listed are present. | All of the elements listed are present. | All of the elements listed are present. | All of the elements listed are present. |
| **Purpose or research question:** Purpose of the study and specific objectives or questions. | The purpose of the study or research question is not clearly stated. | The purpose of the study or research question is clearly stated. | The purpose of the study or research question is clearly stated. | The purpose of the study or research question is not clearly stated. | The purpose of the study or research question is clearly stated. | The purpose of the study or research question is not clearly stated. |
| **Qualitative approach and research paradigm:** Qualitative approach (eg., ethnography, grounded theory, case study, phenomenology, narrative research) and guiding theory if appropriate; identifying the research paradigm (eg., postpositivist, constructivist/ interpretivist) is also recommended; rationale. | The qualitative approach (narrative psychology) and rationale are included, but the research paradigm is not. | Neither a specific qualitative approach nor a research paradigm is provided. | Neither a specific qualitative approach nor a research paradigm is provided. | Neither a specific qualitative approach nor a research paradigm is provided. | The qualitative approach (grounded theory), research paradigm (constructivism), and a rationale are all provided. | Neither a specific qualitative approach nor a research paradigm is provided. |
| **Research characteristics and reflexivity:** Researchers’ characteristics that may influence the research, including personal attributes, qualifications/experience, relationship with participants, assumptions, and/or presuppositions; potential or actual interaction between researchers’ characteristics and the research questions, approach, methods, results, and/or transferability. | The authors mention reflexivity in some way, but do not include any formal reflexivity statement or section. | No mention of reflexivity is made. | No mention of reflexivity is made. | No mention of reflexivity is made. | No mention of reflexivity is made. | No mention of reflexivity is made. |
| **Context:** Setting/site and salient contextual factors; rationale. | The study context and rationale are provided with sufficient detail. | The study context is provided but a rationale is not. | The study context and rationale are provided with sufficient detail. | The study context and rationale are provided but without adequate detail. | The study context is not provided. | The study context and rationale are provided but without adequate detail. |
| **Sampling strategy:** How and why research participants, documents, or events were selected; criteria for deciding when no further sampling was necessary (eg., sampling saturation); rationale. | The sampling strategy is not described. | The sampling strategy is not described. | The sampling strategy is not described. | The sampling strategy is described and a rationale is provided. | The sampling strategy is not described. | The sampling strategy is not described. |
| **Ethical issues pertaining to human subjects:** Documentation of approval by an appropriate ethics review board and participant consent, or explanation for lack thereof; other confidentiality and data security issues. | Documentation of ethics approval is provided (or an explanation for a lack thereof is given). | Documentation of ethics approval is provided (or an explanation for a lack thereof is given). | Documentation of ethics approval is provided (or an explanation for a lack thereof is given). | Documentation of ethics approval is provided (or an explanation for a lack thereof is given). | Documentation of ethics approval is provided (or an explanation for a lack thereof is given). | Documentation of ethics approval is provided (or an explanation for a lack thereof is given). |
| **Data collection methods:** Types of data collected; details of data collection procedures including (as appropriate) start and stop dates of data collection and analysis, iterative process, triangulation of sources/methods, and modification of procedures in response to evolving study findings; rationale. | Data collection methods are described with sufficient detail. | Data collection methods are described with sufficient detail. | Data collection methods are described with sufficient detail. | Data collection methods are described with sufficient detail. | Data collection methods are described with sufficient detail. | Data collection methods are described with sufficient detail. |
| **Data collection instruments and technologies:** Description of instruments (eg., interview guides, questionnaires) and devices (eg., audio recorders) used for data collection; if/how the instrument(s) changed over the course of the study. | Data collection instruments are described with sufficient detail. | Data collection instruments are described with sufficient detail. | Data collection instruments are described with sufficient detail. | Data collection instruments are described with sufficient detail. | Data collection instruments are described with sufficient detail. | Data collection instruments are described but without adequate detail. |
| **Units of study:** Number and relevant characteristics of participants, documents, or events included in the study; level of participation (could be reported in results). | Number and relevant characteristics of participants are provided but without sufficient detail. | Number and relevant characteristics of participants are provided but without sufficient detail. | Number and relevant characteristics of participants are provided but without sufficient detail. | Number and relevant characteristics of participants are provided but without sufficient detail. | Number and relevant characteristics of participants are provided but without sufficient detail. | Number and relevant characteristics of participants are provided but without sufficient detail. |
| **Data processing:** Methods for processing data prior to and during analysis, including transcription, data entry, data management and security, verification of data integrity, data coding, and anonymization/ deidentification of excerpts. | Only some of the elements listed are present. | All of the elements listed are present. | Only some of the elements listed are present. | Only some of the elements listed are present. | None of the elements listed are present. | Only some of the elements listed are present. |
| **Data analysis:** Process by which inferences, themes, etc., were identified and developed, including the researchers involved in data analysis; usually references a specific paradigm or approach; rationale. | Data analysis methods are described with sufficient detail. | Data analysis methods are described with sufficient detail. | Data analysis methods are described with sufficient detail. | Data analysis methods are described with sufficient detail. | Data analysis methods are described with sufficient detail. | Data analysis methods are described with sufficient detail. |
| **Techniques to enhance trustworthiness:** Techniques to enhance trustworthiness and credibility of data analysis (eg., member checking, audit trail, triangulation); rationale. | Techniques to enhance trustworthiness are described but without adequate detail. | Techniques to enhance trustworthiness are described but without adequate detail. | Techniques to enhance trustworthiness are not described. | Techniques to enhance trustworthiness are not described. | Techniques to enhance trustworthiness are described with sufficient detail. | Techniques to enhance trustworthiness are not described. |
| **Synthesis and interpretation:** Main findings (eg., interpretations, inferences, and themes); might include development of a theory or model, or integration with prior research or theory. | Main findings are described with sufficient detail. | Main findings are described with sufficient detail. | Main findings are described with sufficient detail. | Main findings are described with sufficient detail. | Main findings are described with sufficient detail. | Main findings are described with sufficient detail. |
| **Links to empirical data:** Evidence (eg., quotes, field notes, text excerpts, photographs) to substantiate analytic findings. | Links to empirical data are provided. | Links to empirical data are provided. | Links to empirical data are provided. | Links to empirical data are provided. | Links to empirical data are provided. | Links to empirical data are provided. |
| **Integration with prior work, implications, transferability, and contributions to the field:** Short summary of main findings; explanation of how findings and conclusions connect to, support, elaborate on, or challenge conclusions of earlier scholarship; discussion of scope of application/generalizability; identification of unique contribution(s) to scholarship in a discipline or field. | All of the elements listed are present. | All of the elements listed are present. | Only some of the elements listed are present. | All of the elements listed are present. | All of the elements listed are present. | All of the elements listed are present. |
| **Limitations:** Trustworthiness and limitations of findings. | Limitations are listed but no justification or mitigation strategies are provided. | Limitations are listed but no justification or mitigation strategies are provided. | Limitations are listed and justification or mitigation strategies are provided. | Limitations are listed and justification or mitigation strategies are provided. | Limitations are listed but no justification or mitigation strategies are provided. | Limitations are listed but no justification or mitigation strategies are provided. |
| **Conflicts of interest:** Potential sources of influence or perceived influence on study conduct and conclusions; how these were managed. | A conflict of interest statement is provided and no conflicts of interest are disclosed. | Conflicts of interest are disclosed but no indication as to how these were managed is given. | Conflicts of interest are disclosed but no indication as to how these were managed is given. | A conflict of interest statement is provided and no conflicts of interest are disclosed. | A conflict of interest statement is provided and no conflicts of interest are disclosed. | Conflicts of interest are disclosed but no indication as to how these were managed is given. |
| **Funding:** Sources of funding and other support; role of funders in data collection, interpretation, and reporting. | A funding statement is not provided. | Sources of funding support are listed but the role of funders in any aspect of the study is not provided. | Sources of funding support are listed but the role of funders in any aspect of the study are not provided. | No sources of funding support are listed. | Sources of funding support are listed and the role of funders in data collection, interpretation, and/or reporting is provided. | Sources of funding support are listed and the role of funders in data collection, interpretation, and/or reporting is provided. |

**Table S8:** Critical appraisal of studies (part 3).

| **Criterion** | **Sumner et al (2021) [14]** | **Thomson et al (2021) [15]** | **Thomson et al (2023) [16]** | **Watts et al (2017) [17]** |
| --- | --- | --- | --- | --- |
| **Title:** Concise description of the nature and topic of the study identifying the study as qualitative or indicating the approach (eg., ethnography, grounded theory) or data collection methods (eg., interview, focus group) is recommended. | The title identifies the study as qualitative but does not indicate the approach used. | The title neither identifies the study as qualitative nor indicates the approach used. | The title identifies the study as qualitative but does not indicate the approach used. | The title neither identifies the study as qualitative nor indicates the approach used. |
| **Abstract:** Summary of key elements of the study using the abstract format of the intendent publication; typically includes background, purpose, methods, results, and conclusions. | All of the elements listed are present. | All of the elements listed are present. | All of the elements listed are present. | All of the elements listed are present. |
| **Problem formulation:** Description and significance of the problem/phenomenon studied; review of the relevant theory and empirical work; problem statement. | All of the elements listed are present. | All of the elements listed are present. | All of the elements listed are present. | All of the elements listed are present. |
| **Purpose or research question:** Purpose of the study and specific objectives or questions. | The purpose of the study or research question is clearly stated. | The purpose of the study or research question is clearly stated. | The purpose of the study or research question is clearly stated. | The purpose of the study or research question is clearly stated. |
| **Qualitative approach and research paradigm:** Qualitative approach (eg., ethnography, grounded theory, case study, phenomenology, narrative research) and guiding theory if appropriate; identifying the research paradigm (eg. postpositivist, constructivist/ interpretivist) is also recommended; rationale. | Neither a specific qualitative approach nor a research paradigm is provided. | Neither a specific qualitative approach nor a research paradigm is provided. | Neither a specific qualitative approach nor a research paradigm is provided. | The qualitative approach (phenomenology) is provided, but the research paradigm or a rationale are not. |
| **Research characteristics and reflexivity:** Researchers’ characteristics that may influence the research, including personal attributes, qualifications/experience, relationship with participants, assumptions, and/or presuppositions; potential or actual interaction between researchers’ characteristics and the research questions, approach, methods, results, and/or transferability. | No mention of reflexivity is made. | No mention of reflexivity is made. | No mention of reflexivity is made. | No mention of reflexivity is made. |
| **Context:** Setting/site and salient contextual factors; rationale. | The study context and rationale are provided but without adequate detail. | The study context is provided but a rationale is not. | The study context and rationale are provided with sufficient detail. | The study context and rationale are provided with sufficient detail. |
| **Sampling strategy:** How and why research participants, documents, or events were selected; criteria for deciding when no further sampling was necessary (eg., sampling saturation); rationale. | The sampling strategy is not described. | The sampling strategy is described but a rationale is not provided. | The sampling strategy is described and a rationale is provided. | The sampling strategy is not described. |
| **Ethical issues pertaining to human subjects:** Documentation of approval by an appropriate ethics review board and participant consent, or explanation for lack thereof; other confidentiality and data security issues. | Documentation of ethics approval is provided (or an explanation for a lack thereof is given). | Documentation of ethics approval is provided (or an explanation for a lack thereof is given). | Documentation of ethics approval is provided (or an explanation for a lack thereof is given). | Documentation of ethics approval is not provided (with no explanation as to why). |
| **Data collection methods:** Types of data collected; details of data collection procedures including (as appropriate) start and stop dates of data collection and analysis, iterative process, triangulation of sources/methods, and modification of procedures in response to evolving study findings; rationale. | Data collection methods are described with sufficient detail. | Data collection methods are described with sufficient detail. | Data collection methods are described with sufficient detail. | Data collection methods are described with sufficient detail. |
| **Data collection instruments and technologies:** Description of instruments (eg., interview guides, questionnaires) and devices (eg., audio recorders) used for data collection; if/how the instrument(s) changed over the course of the study. | Data collection instruments are described with sufficient detail. | Data collection instruments are described with sufficient detail. | Data collection instruments are described with sufficient detail. | Data collection instruments are described with sufficient detail. |
| **Units of study:** Number and relevant characteristics of participants, documents, or events included in the study; level of participation (could be reported in results). | Number and relevant characteristics of participants are provided but without sufficient detail. | Number and relevant characteristics of participants are provided but without sufficient detail. | Number and relevant characteristics of participants are provided but without sufficient detail. | Number and relevant characteristics of participants are provided but without sufficient detail. |
| **Data processing:** Methods for processing data prior to and during analysis, including transcription, data entry, data management and security, verification of data integrity, data coding, and anonymization/ deidentification of excerpts. | Only some of the elements listed are present. | All of the elements listed are present. | All of the elements listed are present. | Only some of the elements listed are present. |
| **Data analysis:** Process by which inferences, themes, etc., were identified and developed, including the researchers involved in data analysis; usually references a specific paradigm or approach; rationale. | Data analysis methods are described with sufficient detail. | Data analysis methods are described with sufficient detail. | Data analysis methods are described with sufficient detail. | Data analysis methods are described with sufficient detail. |
| **Techniques to enhance trustworthiness:** Techniques to enhance trustworthiness and credibility of data analysis (eg., member checking, audit trail, triangulation); rationale. | Techniques to enhance trustworthiness are described with sufficient detail. | Techniques to enhance trustworthiness are described but without adequate detail. | Techniques to enhance trustworthiness are described with sufficient detail. | Techniques to enhance trustworthiness are described but without adequate detail. |
| **Synthesis and interpretation:** Main findings (eg., interpretations, inferences, and themes); might include development of a theory or model, or integration with prior research or theory. | Main findings are described with sufficient detail. | Main findings are described with sufficient detail. | Main findings are described with sufficient detail. | Main findings are described with sufficient detail. |
| **Links to empirical data:** Evidence (eg., quotes, field notes, text excerpts, photographs) to substantiate analytic findings. | Links to empirical data are provided. | Links to empirical data are provided. | Links to empirical data are provided. | Links to empirical data are provided. |
| **Integration with prior work, implications, transferability, and contributions to the field:** Short summary of main findings; explanation of how findings and conclusions connect to, support, elaborate on, or challenge conclusions of earlier scholarship; discussion of scope of application/generalizability; identification of unique contribution(s) to scholarship in a discipline or field. | All of the elements listed are present. | All of the elements listed are present. | All of the elements listed are present. | All of the elements listed are present. |
| **Limitations:** Trustworthiness and limitations of findings. | Limitations are listed but no justification or mitigation strategies are provided. | Limitations are listed and justification or mitigation strategies are provided. | Limitations are listed and justification or mitigation strategies are provided. | Limitations are listed but no justification or mitigation strategies are provided. |
| **Conflicts of interest:** Potential sources of influence or perceived influence on study conduct and conclusions; how these were managed. | A conflict of interest statement is provided and no conflicts of interest are disclosed. | Conflicts of interest are disclosed but no indication as to how these were managed is given. | Conflicts of interest are disclosed but no indication as to how these were managed is given. | A conflict of interest statement is provided and no conflicts of interest are disclosed. |
| **Funding:** Sources of funding and other support; role of funders in data collection, interpretation, and reporting. | Sources of funding support are listed but the role of funders in any aspect of the study are not provided. | Sources of funding support are listed but the role of funders in any aspect of the study are not provided. | Sources of funding support are listed and the role of funders in data collection, interpretation, and/or reporting is provided. | Sources of funding support are listed but the role of funders in any aspect of the study are not provided. |

**REFERENCES**

1. O’Brien B, Harris I, Beckman T, Reed D, Cook D. Standards for reporting qualitative research: a synthesis of recommendations. Academic Medicine. 2014;89(9).

2. Breeksema J, Niemeijer A, Kuin B, Veraart J, Kamphuis J, Schimmel N, et al. Holding on or letting go? Patient experiences of control, context, and care in oral esketamine treatment for treatment-resistant depression: A qualitative study. Frontiers in Psychiatry. 2022;13.

3. Breeksema J, Niemeijer A, Kuin B, Veraart J, Vermetten E, Kamphuis J, et al. Phenomenology and therapeutic potential of patient experiences during oral esketamine treatment for treatment-resistant depression: an interpretative phenomenological study. Psychopharmacology. 2023;240(7):1547-1560.

4. Breeksema J, Niemeijer A, Krediet E, Karsten T, Kamphuis J, Vermetten E, et al. Patient perspectives and experiences with psilocybin treatment for treatment-resistant depression: a qualitative study. Scientific Reports. 2024;14(1):2929.

5. Griffiths C, Walker K, Reid I, da Silva K, O'Neill-Kerr A. A qualitative study of patients' experience of ketamine treatment for depression: The ‘Ketamine and me’ project. Journal of Affective Disorders Reports. 2021;4:100079.

6. Kerr C, Denee T, Vincent S-A, Bailey KM, Young AH, Rathod S, et al. The lived experience of major and treatment-resistant depression in England: a mixed-methods study. Acta Psychologica. 2023;240:104035.

7. Kragh M, Møller DN, Wihlborg CS, Martiny K, Larsen ER, Videbech P, et al. Experiences of wake and light therapy in patients with depression: a qualitative study. International Journal of Mental Health Nursing. 2017;26(2):170-180.

8. Kroch E, Breheny M, van Kessel K, Taylor J. Order and disorder: Navigating narrative tensions in the experience of treatment resistant depression. Qualitative Psychology. 2021:273-288.

9. Lascelles K, Marzano L, Brand F, Trueman H, McShane R, Hawton K. Effects of ketamine treatment on suicidal ideation: a qualitative study of patients’ accounts following treatment for depression in a UK ketamine clinic. BMJ Open. 2019;9(8):e029108.

10. Lascelles K, Marzano L, Brand F, Trueman H, McShane R, Hawton K. Ketamine treatment for individuals with treatment-resistant depression: longitudinal qualitative interview study of patient experiences. BJPsych Open. 2020;7(1):e9.

11. Lawrence R, Kaufmann C, DeSilva R, Appelbaum P. Patients' beliefs about deep brain stimulation for treatment-resistant depression. AJOB Neuroscience. 2018;9(4):210-218.

12. Raffin Bouchal D, Ferguson A, Green T, McAusland L, Kiss Z, Ramasubbu R. Personal recovery associated with deep brain stimulation for treatment-resistant depression: A constructivist grounded theory study. Journal of psychiatric and mental health nursing. 2023;30(5):1005-1018.

13. Starr H, Abell J, Larish A, Lewis S, DeMuro C, Gogate J, et al. Self-reported review of the value of esketamine in patients with treatment-resistant depression: understanding the patient experience in the STRIVE Study. Psychiatry Research. 2020;293.

14. Sumner R, Chacko E, McMillan R, Spriggs M, Anderson C, Chen J, et al. A qualitative and quantitative account of patient's experiences of ketamine and its antidepressant properties. Journal of Psychopharmacology. 2021;35(8):946-961.

15. Thomson C, Segrave R, Fitzgerald P, Richardson K, Racine E, Carter A. “Nothing to lose, absolutely everything to gain”: patient and caregiver expectations and subjective outcomes of deep brain stimulation for treatment-resistant depression. Frontiers in Human Neuroscience. 2021;15.

16. Thomson C, Segrave R, Fitzgerald P, Richardson K, Racine E, Carter A. Personal and relational changes following deep brain stimulation for treatment-resistant depression: a prospective qualitative study with patients and caregivers. PLoS One. 2023;18(4).

17. Watts R, Day C, Krzanowski J, Nutt D, Carhart-Harris R. Patients’ accounts of increased “connectedness” and “acceptance” after psilocybin for treatment-resistant depression. Journal of Humanistic Psychology. 2017;57(5):520-564.
